# A Drug-Referenced Machine-Learning Framework for Prioritizing Korean Herbal Formulations in Hypertension Using Linear SVM and Graph Convolutional Networks

**DOI:** 10.3390/ijms27198548

**Published:** 2026-09-24

**Authors:** Dong-Woo Lim

**Affiliations:** 1 Department of Diagnostics, College of Korean Medicine, Dongguk University, Goyang 10326, Republic of Korea; greatwoodong@dongguk.edu; Tel.: +82-31-961-5833; 2 Institute of Korean Medicine, Dongguk University, Goyang 10326, Republic of Korea

**Keywords:** hypertension, Korean herbal formulation, network pharmacology, machine learning, graph convolutional network, protein–protein interaction network, drug repurposing, herb–compound–gene network

## Abstract

Hypertension is regulated by interacting molecular systems, providing a rationale for network-based screening of multicomponent therapeutic candidates. Grounded in the conceptual framework of network pharmacology, we developed a hypertension-focused computational framework to prioritize insurance-covered Korean herbal formulations using protein–protein interaction networks derived from marketed antihypertensive and comparator drugs. Thirty reference-drug graphs (15 antihypertensive and 15 comparator drugs) were evaluated using four molecular classifiers under repeated group-aware nested cross-validation. Among them, a linear support vector machine (SVM) integrating protein presence, hypertension-specific disease weight, and normalized degree achieved the highest performance (AUROC, 0.956 ± 0.007) and was applied to rank 56 herbal formulations. Across the 56 formulations, 229 unique representative compounds were retained after deduplication and used to construct formulation-specific molecular networks. The resulting protein-level SVM interpretation identified 110 directionally stable proteins associated with discrimination of the antihypertensive reference set from comparator drugs. No individual protein met strict permutation-calibrated q < 0.05, although eight proteins reached exploratory q < 0.10. Network analysis revealed a 30-protein ion-channel/excitability-associated module with the strongest aggregate model effect (empirical *p* = 0.0002). The highest-ranked formulations showed their greatest stable-signature overlap within a distinct RAAS/vascular receptor-associated module, whereas proteins from the ion-channel/excitability-associated module were sparsely represented across the formulation networks. Blind protein-identity attribution using an identity-aware graph convolutional network provided internal cross-model consistency: 10 of the 15 highest GCN-attributed proteins overlapped the stable SVM signature, compared with 2.58 expected by chance (*p* = 4.78 × 10^−6^), and formed a densely connected candidate subnetwork (empirical *p* = 0.0009). Importantly, the framework is agnostic to the direction of pharmacological action; therefore, high-ranking formulations indicate molecular resemblance to the antihypertensive reference space rather than predicted blood-pressure-lowering efficacy. These findings distinguish a strongly disease-discriminative ion-channel network from a receptor-associated molecular interface more frequently represented within the curated formulation network. The framework provides mechanistically organized hypotheses for experimental validation rather than evidence of clinical antihypertensive efficacy.

## 1. Introduction

Hypertension is one of the most prevalent and consequential modifiable risk factors for cardiovascular, cerebrovascular, and renal disease. An estimated 1.4 billion adults aged 30–79 years worldwide had hypertension in 2024, while only approximately 23% had their blood pressure adequately controlled [1]. Although several effective antihypertensive drug classes are available [2] blood-pressure regulation involves multiple interacting systems, including vascular tone, renal sodium handling, neurohumoral signaling, and the renin–angiotensin–aldosterone system [3]. Large-scale human genetic studies further support this complexity; a recent genome-wide analysis of more than one million individuals identified over 2000 independent blood-pressure-associated genetic signals [4] underscoring the highly polygenic and network-level nature of blood-pressure regulation. This biological complexity provides a rationale for exploring therapeutic candidates that may influence multiple molecular components rather than a single predefined target.

Conventional antihypertensive pharmacotherapy lowers blood pressure through several mechanistically distinct pathways [5]. Calcium-channel blockers reduce vascular smooth-muscle calcium entry and thereby decrease vascular tone [6] whereas angiotensin II receptor blockers inhibit AT1-receptor-mediated actions of the renin–angiotensin system [7]. These well-characterized pharmacological mechanisms provide a clinically grounded molecular reference against which other multicomponent molecular networks can be compared. In the present proof-of-concept study, we therefore use marketed antihypertensive drugs as pharmacological anchors rather than attempting to infer antihypertensive efficacy directly from disease-associated targets.

Multicomponent herbal formulations represent one such source of molecular diversity. Unlike conventional single-compound pharmacology, an herbal prescription may contain numerous constituents associated with multiple proteins and pathways [8]. Systems pharmacology and network-based approaches have therefore been increasingly used to represent herbal medicines as interacting compound–target or protein–protein interaction (PPI) networks [9,10,11]. However, a common limitation of conventional network-pharmacology studies is that they begin with a prespecified formulation, assemble its predicted targets, and subsequently search for disease-related overlap or enriched pathways. Such analyses can be useful for generating mechanistic hypotheses, but target overlap alone does not establish whether a formulation resembles an effective therapeutic intervention. More generally, network-medicine studies suggest that drug action is better understood in relation to the position of drug targets within or near disease-associated network neighborhoods rather than by simple overlap with isolated disease genes [12,13,14]. A framework that first learns the molecular characteristics of established therapeutic drugs and subsequently applies this learned representation to candidate formulations may therefore provide a complementary and less candidate-centered strategy for prioritization [15].

Machine-learning models offer a practical means of implementing such a reference-drug-based approach. Protein composition, disease relevance, and local network topology can be encoded as high-dimensional features for conventional classifiers, whereas graph neural networks can additionally incorporate explicit PPI connectivity [16,17]. Importantly, greater model complexity does not necessarily guarantee better prediction when the number of independent labeled biological samples is limited [18]. For small drug-network datasets, a transparent linear model may provide a stable primary classifier, while a graph-based model can offer complementary information about the molecular context underlying individual predictions. This combination is particularly attractive for multicomponent formulations, for which the objective is not only to generate a ranking but also to determine whether highly ranked candidates converge on coherent molecular neighborhoods. Such an approach also allows protein-level effects to be evaluated at the network or module level, which is relevant given the strongly interconnected architecture of blood-pressure regulation.

In the present study, we developed an HTN-focused computational framework using drug-specific PPI networks from marketed antihypertensive and comparator drugs as the reference space. Four molecular classifiers—protein-presence logistic regression, HTN-weighted logistic regression, a protein-specific linear support vector machine (SVM), and an identity-aware graph convolutional network (GCN)—were evaluated under repeated group-aware nested cross-validation. The best-performing classifier was then applied to a fixed library of 56 insurance-covered Korean herbal formulations [19,20] to generate a model-based prioritization. Rather than interpreting ranking alone, we subsequently decomposed the primary model at the protein level, evaluated the resulting antihypertensive reference-associated signature using repeated out-of-fold contributions and label-permutation calibration, and examined its organization within PPI modules. We then quantified how the prioritized formulations intersected these molecular modules and used blind GCN protein-identity attribution as a secondary analysis of cross-model mechanistic convergence. The objective was therefore not to infer clinical antihypertensive efficacy directly, but to establish a reproducible framework for identifying herbal formulation networks that most closely converge with the molecular architecture learned from established antihypertensive pharmacology and to generate experimentally testable mechanistic hypotheses.

## 2. Results

### 2.1. Construction and Quality Control of the HTN Reference Network Dataset

Following data curation, the final HTN reference dataset comprised 30 drug-specific PPI graphs, including 15 antihypertensive drugs and 15 comparator drugs. Across the 30 graphs, 1254 unique protein identities, 2257 protein nodes, and 14,430 PPI edges were represented. The median graph contained 77.5 nodes and 346 edges, and the mean proportion of nodes with available HTN-specific disease weights was 0.462. Network characteristics of the individual reference drugs are provided in Table S1.

### 2.2. The Linear SVM Showed the Highest HTN Classification Performance

Among the four evaluated models, the linear SVM achieved the highest mean AUROC and showed the lowest cross-seed variability (Figure 1A,B and Table 1), with an AUROC of 0.956 ± 0.007, accuracy of 0.940 ± 0.014, F1 score of 0.944 ± 0.013, and MCC of 0.887 ± 0.026. The identity-aware GCN achieved an AUROC of 0.936 ± 0.033, accuracy of 0.853 ± 0.063, F1 score of 0.860 ± 0.058, and MCC of 0.714 ± 0.120. Presence-LR and weighted-presence LR yielded AUROCs of 0.899 ± 0.048 and 0.895 ± 0.034, respectively. The same model ordering was observed across all four evaluation metrics (Figure 1C). The linear SVM was selected as the primary ranking model based on its highest mean discrimination, lower cross-seed variability than the GCN (AUROC SD, 0.007 vs. 0.033), and the direct decomposability of its decision function into interpretable protein-level feature contributions. Seed-wise out-of-fold performance and coefficient-reproduction quality control for the primary SVM are summarized in Table S2.

In a post hoc GCN sensitivity analysis, removal of protein-specific identity embeddings reduced the repeated-seed AUROC from 0.936 ± 0.033 to 0.707 ± 0.079. A vocabulary-status control retaining only known-versus-OOV identity information yielded an AUROC of 0.668 ± 0.095, indicating that binary vocabulary-recognition status alone did not reproduce the discrimination of the full identity-aware model (Figure S1).

Class-held-out analysis showed substantial cross-class transfer. Training on CCBs and evaluating unseen ARBs yielded a mean AUROC of 0.849 ± 0.073, whereas training on ARBs and evaluating unseen CCBs yielded 0.785 ± 0.080 (Figure S2).

To assess potential circularity introduced by this disease-specific weighting, we additionally performed a sensitivity analysis. Removal of the GeneCards hypertension-weight channel produced only a small reduction in mean AUROC (0.956 ± 0.007 to 0.952 ± 0.005). The resulting stable signature contained 108 proteins, including 107 of the original 110 proteins (97.3%; Jaccard similarity = 0.964), and formulation rankings remained highly concordant (Spearman ρ = 0.990), with complete preservation of the original top 10 formulations (Figure S3). Individual out-of-fold predictions for all 30 reference drugs are additionally provided, summarized as the mean decision score across the 10 repeated seeds with the corresponding seed-level dispersion; discrimination based on these drug-level mean scores was AUROC 0.951 (bootstrap 95% CI 0.840–1.000) (Figure S4). Separation was consistent across the antihypertensive classes, although individual drugs differed in the stability of their predictions across seeds.

### 2.3. Repeated SVM Screening Identified a Highly Stable Set of Prioritized Herbal Formulations

The 50 outer-fold SVM models were applied to all 56 insurance-covered Korean herbal formulations (Figure 2A). Daehwangmokdanpi-tang and Doinseunggi-tang ranked first and second, followed by Daecheongryong-tang, Hyangsapyeongwi-san, Haengso-tang, Sigyeongbanha-tang, and Antae-eum. These seven formulations appeared within the Top 10 in all 50 SVM models. Daehwajung-eum and Naeso-san appeared within the Top 10 in 49 of 50 models, and Samchulgeonbi-tang in 47 of 50 models. The corresponding rank distributions across the 50 models are shown in Figure 2B.

The leading SVM-ranked formulations also generally received high rankings from the secondary models, although their relative ordering differed across model representations (Figure 2C).

To assess whether the formulation ranking reflected learned molecular features rather than the upstream protein-selection rule or graph size, the 56 formulations were re-scored under the existing 50 outer-fold SVM models without refitting. Reconstructing candidate graphs under harmonized STITCH confidence floors (c = 0.300–0.700) reproduced the original ranking (Spearman ρ = 0.997–1.000; Top 10 fully retained), while a size-restricted design combining a common confidence floor (c = 0.300) with an upper limit of 78 proteins also retained all top-10 formulations (ρ = 0.876). Re-ranking by mean contribution per recognized protein and balanced subsampling to a common number of recognized proteins likewise preserved the ranking (ρ = 0.956 and 0.955, respectively; top 10 fully retained), and removal of the 18 vocabulary proteins shared across the Top 10 did not alter the ordering (ρ = 0.999). Applying the identical formulation graphs to a type 2 diabetes reference model yielded an entirely different Top 10 (overlap 0/10; ρ = 0.506), whereas a hyperlipidemia model produced a partially overlapping ranking (7/10; ρ = 0.901). Full results are provided in Tables S3 and S4.

### 2.4. Protein-Level SVM Interpretation Revealed a Reproducible HTN-Associated Molecular Pattern

Protein-level interpretation of the linear SVM identified recurrent positive contributions from proteins including REN, SCN5A, SCN3A, CACNA2D1, AGTR1, CACNA1S, CACNA1D, CACNA1C, and AGTR2 (Figure 3A).

Of these 20 most strongly drug-discriminative proteins, only AGTR2 and CCR3 were represented in the prioritized formulation networks; the remaining 18, comprising the calcium- and sodium-channel proteins together with REN, ACE, and AGTR1, were absent and therefore contributed exactly zero to the formulation scores (Figure 3B).

This did not reflect a sparse contribution profile. Each of the five representative formulations contained 36–48 proteins within the model vocabulary, and AGTR2 and CCR3 together accounted for only 14–17% of the total positive contribution to the decision score. Decomposition of the decision function identified 32 proteins with a mean absolute contribution above 0.002, of which 24 contributed positively and 8 negatively (Figure 3C). The positive contributors were predominantly receptor-associated proteins, led by AGTR2, CNR2, CCR3, ADORA3, and GPR55, whereas HSD11B1, NR3C1, GRM2, and GRM3 contributed in the opposite direction. Individual formulation values were closely aligned across the five networks, indicating a reproducible multicomponent contribution profile rather than dependence on a small number of proteins.

### 2.5. Permutation Calibration Supported a Stable Multicomponent Signature but Not Strict Single-Protein Significance

A total of 110 proteins satisfied the predefined directional-stability criteria based on repeated out-of-fold SVM contributions (Figure 4A and Table S5). REN showed the largest HTN-versus-comparator OOF contribution difference, followed by SCN5A, SCN3A, CACNA2D1, CACNA1S, AGTR1, CACNA1C, and CACNA1D.

After 1000 label permutations, no individual protein met the strict empirical q < 0.05 threshold. The minimum empirical q-value was 0.099, and eight proteins—REN, SCN5A, SCN3A, CACNA2D1, CACNA1S, AGTR1, CACNA1C, and CACNA1D—met the exploratory q < 0.10 threshold.

### 2.6. Stable SVM Proteins Formed Six PPI Network Modules

Projection of the 110-protein stable SVM signature onto the antihypertensive-drug PPI networks identified six network modules (Figure 4B,C and Table 2). M1 was the largest module, comprising 57 proteins and 269 edges, with a network density of 0.169 and a mean OOF contribution difference of 0.00425. This module contained REN, ACE, AGT, AGTR1, AGTR2, EDNRB, CCR3, and other receptor-associated proteins and was descriptively annotated as a RAAS/vascular receptor-associated module. Its aggregate effect did not exceed that of same-size random stable-protein sets (P_emp_ = 0.987).

M2 comprised 30 proteins and 235 edges and had a higher network density of 0.540. It included multiple sodium- and calcium-channel proteins, including SCN5A, SCN3A, CACNA1C, CACNA1D, CACNA1S, and CACNA2D1. M2 showed the highest module-level mean OOF contribution difference (0.00729) and exceeded 5000 same-size random protein sets (P_emp_ = 0.00020). The remaining four smaller modules did not show significant aggregate effects.

### 2.7. Prioritized Formulations Showed Greater Coverage of the M1 Module

Weighted coverage of the 110-protein stable SVM-derived antihypertensive-reference-associated signature among the top-ranked formulations ranged from approximately 10.5% to 15.2% (Figure 5A and Table 3). Daecheongryong-tang showed the highest coverage (15.16%; 22 stable proteins), followed by Daehwangmokdanpi-tang, Doinseunggi-tang, and Hyangsapyeongwi-san (14.87%; 21 proteins each). Haengso-tang, Sigyeongbanha-tang, and Antae-eum each showed approximately 14.57% weighted coverage.

Module-specific analysis showed that the leading formulations predominantly overlapped with M1 (Figure 5A). Daecheongryong-tang, Daehwangmokdanpi-tang, Doinseunggi-tang, and Hyangsapyeongwi-san each captured approximately 34% of the weighted M1 signal, whereas M2 coverage was minimal. Among these leading formulations, only Daecheongryong-tang contained an M2-associated protein, TRPA1. At the individual-protein level, the captured members of the stable signature were predominantly receptor-associated proteins, including AGTR2, CCR3, AGT, CNR2, CXCR4, and multiple neuropeptide and lysophosphatidic acid receptors (Figure 5B). Across the full formulation library, the highest-ranked formulations generally showed greater weighted stable-signature coverage than lower-ranked formulations (Figure 5C and Table S6). The apparent difference around the Top-10 boundary was treated descriptively and was not interpreted as a biological threshold.

### 2.8. GCN Attribution Overlapped with the SVM M1 Signature and Formed a Coherent PPI Subnetwork

Blind protein-identity attribution was performed using the identity-aware GCN for Antae-eum, Hyangsapyeongwi-san, and Sigyeongbanha-tang (Figure 6A). Among the GCN Top-15 attributed proteins, 10 overlapped with the stable SVM signature, and all 10 belonged to SVM Module 1. Within the 122-protein union of the three formulation networks, 21 proteins belonged to the stable SVM signature; the observed overlap of 10 proteins exceeded the random expectation of 2.58 (exact hypergeometric *p* = 4.78 × 10^−6^; Figure 6B).

GCN attribution magnitude showed only a modest, non-significant correlation with the corresponding SVM OOF protein effects (ρ = 0.364, *p* = 0.182). The GCN top-15 proteins nevertheless formed a single connected PPI component containing 45 edges, compared with a mean of 16.94 edges in 10,000 random 15-protein sets from the same candidate-protein universe (empirical *p* = 0.0009; Figure 6C). A summary of all cross-model convergence analyses, including the shared and GCN-attributed-only protein sets, is presented in Table 4. Decoded protein identities, attribution magnitudes, and cross-model metrics for the GCN Top-15 set are provided in Table S7.

### 2.9. The Formulation Ranking Corresponded to Independently Measured Vasorelaxant Activity

Twenty-two of the 56 formulations had previously been assayed for vasorelaxant activity on isolated rat aortic rings in an independent published screen of 50 prescriptions [21]. The mean SVM decision score correlated with the reported relaxation at 1000 µg/mL (Spearman ρ = 0.525, *p* = 0.0122; permutation *p* = 0.0142; Figure S5 and Table S8). The correlation remained significant in all 22 leave-one-out analyses (ρ = 0.461–0.628) and after exclusion of the single extreme observation (ρ = 0.497, *p* = 0.0219). The two prescriptions reported to produce net vasoconstriction in that dataset, Sosiho-tang and Samsoeum, ranked 40th and 48th of 56 in the present model.

## 3. Discussion

In this study, we developed a hypertension-focused molecular screening framework that used marketed antihypertensive and comparator drug networks as a reference space for prioritizing insurance-covered Korean herbal formulations. Among the four evaluated models, the linear SVM achieved the highest repeated cross-validated performance and was therefore used as the primary ranking model, whereas the identity-aware GCN was retained as a complementary graph-based model. Protein-level analysis identified a stable multicomponent signature that discriminated the current antihypertensive reference drugs from the comparator-drug set. This signature was further organized into distinct network modules. Because the positive reference set consisted of CCBs and ARBs, this signature represents the molecular structure of the defined antihypertensive reference space rather than a comprehensive signature of hypertension pharmacology. Nevertheless, class-held-out transfer analysis showed that a model trained on CCBs retained discrimination of unseen ARBs, and vice versa, with mean AUROCs of 0.849 and 0.785, respectively. This cross-class transfer suggests that the learned representation is not reducible solely to within-class recognition of CCB- or ARB-specific molecular patterns, although generalization beyond these two pharmacological classes remains untested. Notably, the strongest aggregate SVM signal was concentrated in an ion-channel/excitability-associated module (M2), whereas the highest-ranked herbal formulations predominantly overlapped with a receptor-rich module (M1). The secondary GCN analysis also preferentially emphasized proteins within M1. These findings suggest that the molecular features most strongly distinguishing established antihypertensive drugs are not necessarily identical to those most extensively represented by multicomponent herbal formulations.

The prominence of the M2 ion-channel module is consistent with established blood-pressure biology. Calcium-channel proteins are biologically compatible with hypertension pharmacology, but their prominence is also expected to reflect the substantial representation of calcium-channel blockers in the positive reference set. Large-scale genetic studies have implicated vascular-function and ion-transport pathways in blood-pressure regulation and hypertension [4,22,23]. Several calcium-channel proteins identified in the present signature, including CACNA1C, CACNA1D, CACNA1S, CACNA2D1, and CACNB2 [24] are compatible with the central role of calcium-dependent excitation–contraction coupling in vascular smooth muscle [25] and with the established antihypertensive effects of L-type calcium-channel blockade [26]. Multiple voltage-gated sodium-channel proteins were also represented. Although these channels are less directly associated with conventional antihypertensive therapy, sodium currents have been described in vascular smooth muscle and may contribute to membrane excitability and vascular tone [27]. M2 should therefore be interpreted as an ion-channel/excitability-associated network rather than as evidence that each constituent protein represents an independent therapeutic target. Because HTN-specific disease weights were incorporated into the model, recovery of established hypertension-related biology represents biological consistency of the learned signature rather than a completely prior-free discovery.

The prioritized formulations showed a different pattern, with substantially greater representation of the receptor-rich M1 module. M1 was defined within the reference-drug space and contained canonical RAAS proteins (AGT, REN, ACE, AGTR1) together with receptor- and neurohumoral-signaling proteins including AGTR2, CCR3, CNR2, CXCR4, and EDNRB [28]. The relevance of RAAS signaling to hypertension is well established genetically and pharmacologically [29,30]. However, M1 did not show an unusually large aggregate SVM effect in the random-set analysis and should not be interpreted as the statistically strongest HTN module. Rather, its importance in the present study arose from its recurrent representation within the prioritized formulation networks. Notably, the M1 proteins recurrently represented in those networks were the receptor-associated members—AGTR2, CCR3, CNR2, CXCR4, and AGT—whereas ACE and AGTR1 were not represented in any of the 56 formulation networks, and REN appeared only in lower-ranked formulations (Figure 5B). One possible interpretation is that M2 represents a stronger drug-class-discriminative component of the reference space, whereas M1 represents a molecular region more extensively accessible to the broader target profiles of multicomponent formulations. This interpretation is consistent with network-medicine concepts in which therapeutic perturbations may act through disease-relevant network neighborhoods rather than exclusively through the most strongly disease-associated individual proteins [13,14,15]. Accordingly, M1 coverage by the prioritized formulations reflects engagement with a receptor-associated periphery of the RAAS module rather than with its canonical RAAS axis, and therefore provides a mechanistic hypothesis for formulation prioritization without demonstrating direct regulation of the RAAS or antihypertensive efficacy.

The absence of ACE, AGTR1, and EDNRB from the formulation networks should not be interpreted as evidence that these pathways are biologically irrelevant to the formulations. Rather, formulation-level target representation is conditional on the selected marker compounds, STITCH database coverage, and the top-100 target-retention rule. Accordingly, the observed M1 coverage reflects the molecular representation available within the present network-construction framework. Conversely, several blood-pressure-relevant proteins were widely represented across the formulation networks, including HSD11B2 and NR3C2 (43 of 56 formulations each)—consistent with the established mineralocorticoid effects of licorice-derived glycyrrhizin—as well as NOS3 (35 of 56). These targets were, however, less frequently retained in the highest-ranked formulations, whose 100-protein lists were truncated at a STITCH score of 0.900; the biological content of the top-ranked networks is therefore narrower than the underlying chemistry would support.

The exploratory GCN analysis provided an additional perspective on cross-model consistency at the network-level interpretation. Ten of the 15 highest GCN-attributed protein identities were also members of the stable SVM signature, and all 10 belonged to M1; this overlap was greater than expected by chance (*p* = 4.78 × 10^−6^). The GCN-attributed proteins also formed a highly connected PPI subnetwork (P_emp_ = 0.0009). In contrast, the correlation between individual GCN attribution magnitudes and SVM protein effects was modest, indicating that the two architectures did not reproduce the same protein-level ranking. Thus, the principal cross-model agreement occurred at the level of a shared molecular neighborhood rather than exact correspondence of individual protein importance. Because the SVM and GCN were derived from overlapping molecular source data, this agreement should be interpreted as internal cross-model consistency rather than independent biological validation. Moreover, the GCN attribution analysis was exploratory and restricted to three selected formulations, which limits the generalizability of the cross-model comparison. GCN-specific attributed proteins may provide additional hypotheses for subsequent mechanistic investigation, but their model attribution does not establish pharmacological activity.

The prevalence of OOV proteins differed between the antihypertensive and comparator reference classes, indicating that vocabulary coverage may itself carry some class information. However, a control GCN retaining only known-versus-OOV status performed substantially worse than the full identity-aware model, suggesting that this shortcut alone does not account for the observed GCN discrimination; nevertheless, the influence of reference-set composition cannot be completely excluded.

The greater coverage of M1 by the prioritized formulations should not be interpreted as evidence that M1 is biologically more relevant to hypertension than M2. Rather, it suggests that the represented chemical–target space of these formulations is more frequently connected to receptor-associated proteins within M1, whereas proteins belonging to the ion-channel/excitability-associated M2 signature are sparsely represented in the formulation networks. This difference therefore reflects the molecular representation accessible through the curated compounds and database-derived compound–protein associations, rather than a biological hierarchy between the two modules.

Although the present framework was not validated experimentally, several of the highest-ranked formulations have been examined pharmacologically elsewhere. Samchulgeonbi-tang lowered systolic blood pressure and restored endothelium-dependent relaxation with increased eNOS phosphorylation in hypertensive rats [31]. Doinseunggi-tang suppressed vascular contraction and enhanced relaxation in isolated vessels under oxidative-stress loading [32] and increased erythrocyte velocity and flow volume from arteries through arterioles to capillaries in live imaging [33]; together with Daehwangmokdanpi-tang, it also improved peripheral microcirculatory flow in a blood-stasis model [34]. We do not present this survey as validation, and we deliberately refrain from testing whether prior evidence is enriched among the highest-ranked formulations. The published literature is concentrated on formulations already suspected of cardiovascular activity, so the presence of a study reflects prior investigator interest as much as pharmacology, and its absence is not evidence of inactivity. A comparison with vasorelaxant measurements obtained independently of this work showed a significant correlation with the model ranking (ρ = 0.525, *p* = 0.0122), although that endpoint is ex vivo vascular relaxation rather than blood pressure and covers 22 of the 56 formulations (Figure S5, Table S8).

Several limitations should be considered. First, the reference dataset contained only 30 drug graphs, and repeated cross-validation assessed split stability without increasing the underlying biological sample size [18,35]. Second, the positive reference set represents marketed antihypertensive pharmacology—specifically calcium-channel blockade and angiotensin receptor blockade —rather than the complete pathophysiology of human hypertension; the resulting model therefore identifies an antihypertensive-drug-like molecular pattern primarily. Third, HTN-specific disease weights were incorporated as input features, so biological interpretation is partly informed by prior disease knowledge. Fourth, no individual protein met the strict permutation-calibrated q < 0.05 thresholds, and the mechanistic interpretation therefore relies principally on reproducible multivariate patterns and network-level organization. Fifth, the target and PPI representations do not capture constituent concentration, bioavailability, tissue exposure, direction of pharmacological action [36] or herb–drug interactions [37,38]. Daecheongryong-tang, the formulation with the highest weighted signature coverage, illustrates this directly: it contains Ephedra Herba as its principal constituent, and ephedrine-type alkaloids act as sympathomimetic agents that raise rather than lower blood pressure [39], indicating that a high model rank denotes engagement with antihypertensive-reference target space rather than a predicted direction of blood-pressure change. Beyond the external ex vivo comparison reported here, the prioritized formulations have not been validated in an in vivo experimental hypertension model or clinical cohort, and the predicted molecular interactions have not been directly experimentally confirmed.

A further limitation is the use of five representative markers or major compounds per herb. Although these compounds were selected a priori from curated pharmacognostic sources, they do not represent the complete phytochemical or pharmacologically active constituent space of each herb. Minor constituents, relative abundance, bioavailability, and tissue exposure were not incorporated quantitatively. Accordingly, the resulting formulation networks should be interpreted as standardized molecular representations rather than exhaustive chemical profiles.

A fundamental boundary of the present framework is that target engagement does not imply therapeutic direction. Although the linear SVM itself is not globally monotonic, because individual protein features may contribute positively or negatively to the decision function, the underlying compound–protein associations are unsigned and do not distinguish agonism from antagonism, activation from inhibition, or other directionally opposing pharmacological effects. Thus, pharmacologically opposite interactions with the same protein may be represented as engagement of the same molecular feature. Accordingly, a formulation may overlap substantially with proteins that discriminate antihypertensive drugs while producing effects at those proteins that differ from, or even oppose, those of the reference drugs. The resulting rankings should therefore be interpreted as measures of molecular resemblance to the antihypertensive reference space rather than as predictions of antihypertensive efficacy or the direction of blood-pressure change. Experimental pharmacological validation would be required to establish the direction and net physiological consequences of these molecular interactions.

The restricted pharmacological composition of the positive reference set was also related to the proof-of-concept design of this study. Rather than combining numerous mechanistically distinct antihypertensive classes represented by only a few drugs each, the initial framework used two established and internally interpretable pharmacological classes, CCBs and ARBs, to define a relatively coherent therapeutic reference space. In a small graph-level dataset, excessive mechanistic heterogeneity may obscure reproducible class-discriminative molecular structure and complicate biological interpretation. The resulting model should therefore be understood as learning the molecular architecture of this defined antihypertensive reference space, rather than a universal molecular representation of hypertension treatment. Extension to ACE inhibitors, β-blockers, thiazide diuretics, mineralocorticoid receptor antagonists, and other classes will be necessary to evaluate how the learned signature and formulation ranking change as the reference space becomes pharmacologically broader.

The present results should therefore be regarded as computational prioritization and hypothesis generation rather than evidence of clinical antihypertensive efficacy. Future studies should combine standardized chemical characterization with vascular and pathway-specific experimental assays and validation in appropriate hypertension models.

## 4. Materials and Methods

### 4.1. Study Design and Analytical Framework

This study developed a hypertension (HTN)-focused computational framework to prioritize insurance-covered Korean herbal formulations and to investigate the molecular network features underlying their model-based prioritization. The analytical workflow comprised five sequential stages: (1) construction and quality control of drug-specific protein–protein interaction (PPI) networks for marketed antihypertensive and comparator drugs; (2) repeated group-aware cross-validation of four molecular classification models; (3) prioritization of 56 insurance-covered Korean herbal formulations using the best-performing classifier; (4) protein- and network-level interpretation of the antihypertensive-reference-associated signature; and (5) cross-model mechanistic analysis using protein-identity attribution from an identity-aware GCN (Figure 7).

The primary predictive model was selected based on repeated out-of-fold discrimination, cross-seed stability, and interpretability. The linear SVM served as the primary classifier, whereas the identity-aware GCN was retained as a graph-structured secondary model for complementary mechanistic interpretation. Formulation scores were interpreted as computational prioritization measures and not as calibrated probabilities of therapeutic efficacy.

### 4.2. Hypertension Reference-Drug Dataset

The reference-drug dataset used in this study was derived from marketed-drug information collected through the Health Insurance Review & Assessment Service (HIRA, Republic of Korea; https://www.hira.or.kr/ accessed 15 January 2024). Drugs indicated for HTN were assigned the positive label (y = 1), whereas drugs used for other indications and not designated as antihypertensive agents were used as comparator drugs and assigned the negative label (y = 0). Comparator drugs were selected from the curated marketed-drug pool on the basis of the absence of an antihypertensive indication and availability of sufficient molecular association data to construct a valid drug-specific network. The resulting comparator set spanned multiple therapeutic categories and was intended to provide a heterogeneous marketed-drug reference class rather than a mechanistically homogeneous negative-control group. Drug identities, representative indications, therapeutic categories, and network characteristics are provided in Table S1. The comparator drugs were not intended to represent biologically HTN-negative controls. Rather, they provided a marketed-drug reference class for distinguishing molecular features preferentially associated with established antihypertensive pharmacology from features broadly represented across drug-associated PPI networks.

The HTN reference dataset contained 30 marketed-drug graphs, comprising 15 antihypertensive drugs and 15 comparator drugs. Across these graphs, 1254 unique protein identities were represented, corresponding to 2257 total protein nodes and 14,430 protein–protein interaction (PPI) edges (Table 5).

The final positive set comprised calcium-channel blockers (*n* = 9) and angiotensin receptor blockers (*n* = 6), the two classes that dominate first-line antihypertensive prescribing in Korea, where angiotensin receptor blockers and calcium-channel blockers account for 51.6% and 45.0% of prescriptions in treatment-naive patients compared with 11.7% for ACE inhibitors [40]. This composition was chosen to reflect the pharmacological context in which the insurance-covered herbal formulations under study are actually prescribed.

### 4.3. Construction and Quality Control of Compound–Protein and Protein–Protein Interaction Networks

Chemical–protein associations used to define the protein sets of the reference drugs and herbal formulations were obtained from the Search Tool for Interactions of Chemicals (STITCH, version 5.0) [41] and the resulting protein lists were used to construct human PPI networks using the Search Tool for the Retrieval of Interacting Genes/Proteins (STRING) version 12.0 [42].

For marketed drugs, drug-associated proteins were retrieved directly from STITCH. For the insurance-covered herbal formulations, constituent-herb information for the 56 reimbursed prescriptions was obtained from the Price List of Standard Prescription for Herbal Health Insurance provided by the National Health Insurance Service. Across the 56 formulations, 67 distinct constituent herbs were identified.

Representative compound information for these herbs was compiled by integrating data from the Korean Pharmacopoeia, the Korean Medicine Convergence Research Information Center (KMCRIC), and the Herbal Medicine Quality Standardization Research Program [43,44,45]. Compound information was available for 62 of the 67 herbs, whereas corresponding information was unavailable for the remaining five herbs. Five representative marker or major compounds were selected a priori for each herb from the integrated source information in References [43,44,45]. Compound selection was independent of STITCH target profiles, model outputs, or formulation rankings. These compounds were used as a standardized molecular representation of each herb and were not intended to capture its complete phytochemical or pharmacologically active constituent spectrum. After removal of compounds shared across multiple herbs, 229 unique compounds were retained. These compounds were queried in STITCH, and for each formulation the associated proteins were retained in descending order of the STITCH combined score up to a maximum of 100 proteins per formulation. No fixed confidence threshold was applied; consequently, the minimum retained score varied across formulations (range, 0.237–0.900; median, 0.786), and eight of the 56 formulations reached the 100-protein limit. Because this rule determines graph membership, STITCH scores influenced network construction upstream of model fitting even though they were not used as continuous predictive features. Sensitivity analyses using harmonized confidence thresholds are reported in Table S3.

The final machine-learning models did not use STITCH chemical–protein interaction scores or STRING confidence scores as continuous predictive features. Rather, the resulting protein identities and binary PPI connectivity were used to construct the model input graphs. Compound–protein associations were represented as unsigned molecular associations; information regarding agonism versus antagonism, activation versus inhibition, or other directional pharmacological effects was not incorporated into the network representation.

STRING interactions were retained and converted to binary, undirected adjacency matrices. An edge value of 1 indicated the presence of an interaction between two proteins and 0 indicated its absence; the original STRING confidence value was not retained as a continuous edge weight. The same network-construction procedure was applied to marketed drugs and the 56 insurance-covered Korean herbal formulations to maintain a common molecular representation across the reference and candidate datasets.

All resulting adjacency matrices underwent structural quality control before modeling. Each matrix was required to be square, binary, and symmetric. Protein identifiers and their ordering were to be identical between the row and column labels, and all diagonal entries were required to be zero, indicating that computational self-connections were not included in the PPI metrics. The correspondence between protein identities and node-level feature data was also verified. The same structural quality-control procedure was applied to both the reference drugs and 56 herbal formulation graphs.

### 4.4. HTN-Specific Protein Weights and Protein Identity Mapping

HTN-specific protein relevance scores were obtained from GeneCards (version 5.19) using “Hypertension” as the disease query [46]. The GeneCards hypertension score was incorporated as an auxiliary disease-relevance prior to modulate protein-level features rather than as a defining criterion for protein inclusion. The original GeneCards relevance scores were standardized by z-score normalization and subsequently transformed using a sigmoid function. The resulting values were mapped to the encoded protein identities and used as HTN-specific node-level disease weights. Proteins without a mapped weight were assigned a value of zero and distinguished using a separate weight-availability indicator. The mean HTN-weight mapping ratio across the final 30 reference-drug graphs was 0.462 (Table 5).

For the final identity-aware GCN, each protein node was characterized by its protein identity and associated numerical information. Proteins represented in the outer-training vocabulary were assigned trainable identity embeddings. Proteins absent from the outer-training vocabulary were mapped to a shared out-of-vocabulary (OOV) index (index 0), implemented as a fixed zero embedding (padding_idx = 0); the OOV embedding was therefore not updated during training. The numerical node features included HTN-specific disease weight, an indicator of disease-weight availability, normalized node degree, and a constant feature.

### 4.5. Molecular Classification Models

Four molecular classification models were evaluated using identical seed-specific outer cross-validation assignments.

(1) Presence-LR. An elastic-net logistic regression model was fitted using binary indicators of protein presence. Each feature indicated whether a protein from the outer-training vocabulary was present in a given graph.
(2) Weighted-presence LR. The second elastic-net logistic regression model used the HTN disease weight for each present protein; absent proteins and proteins without a mapped disease weight were assigned zero. Elastic-net logistic regression was implemented using SGDClassifier with logistic loss, an elastic-net penalty, and l1_ratio = 0.5 [47]. The regularization parameter alpha was selected from 10^−4^, 10^−3^, and 10^−2^ using inner cross-validation.
(3) Linear SVM. The primary linear SVM representation contained three protein-specific channels: binary protein presence, HTN disease weight, and normalized graph degree. Thus, a protein represented in the outer-training vocabulary contributed three corresponding features. Features were standardized using StandardScaler (with_mean = False), followed by LinearSVC with class-weight balancing [48]. The regularization parameter C was selected from 0.1, 1, and 10 within each outer-training partition.
(4) Identity-aware GCN. The GCN incorporated a learnable protein-identity embedding, numerical node features, and the full binary drug-specific PPI adjacency matrix. For GCN message passing, self-loops were added computationally to the adjacency matrix, followed by symmetric degree normalization [49]. The HTN GCN used a fixed two-layer residual architecture with a hidden dimension of 16, combined sum and mean graph pooling, a dropout rate of 0.25 [50], a learning rate of 0.003, and a weight decay of 0.001. This architecture was fixed before the final repeated-seed validation, with inner validation used only for epoch selection.

For each node, the learnable protein embedding and projected numerical features were combined before graph propagation. At each graph-convolution step, normalized adjacency-based message passing was followed by a linear transformation, rectified linear activation, residual addition, and dropout [51]. Final node representations were separately summed and averaged over the graph; the two pooled vectors were concatenated and passed to a binary graph-level classifier. Training used the Adam optimizer and binary cross-entropy with logits [52].

### 4.6. Repeated Group-Aware Nested Cross-Validation

Grouping was defined by graph identity: each drug graph was assigned a hash of its protein composition and adjacency structure, and StratifiedGroupKFold was applied with these hashes as the grouping variable, so that graphs with identical composition could not be divided across training and test partitions while class balance was preserved within each fold. In the final curated dataset, every one of the thirty graphs carried a distinct hash, so each drug formed its own group; the procedure therefore acted as a structural safeguard rather than merging any drugs. Inner three-fold cross-validation used the same grouping.

Model performance was evaluated using stratified, group-aware five-fold outer cross-validation repeated across 10 predetermined random seeds, yielding 50 outer-fold evaluations per model [35]. The same seed-specific outer-fold assignments were used for all models to ensure a consistent comparison.

Protein-specific feature vocabularies were constructed exclusively from the corresponding outer-training partition. Proteins occurring only in an outer-test graph were therefore not introduced into the training vocabulary. For the identity-aware GCN, such proteins were represented by a shared OOV identity while retaining their numerical node features and PPI connectivity. The shared OOV identity was represented by this fixed zero embedding rather than by a trainable OOV parameter. As a post hoc sensitivity analysis, the contribution of protein-identity encoding and the potential influence of known-versus-OOV vocabulary status were evaluated using identity-free and vocabulary-status control GCNs under the same repeated outer-validation framework.

For the conventional machine-learning models, hyperparameters were selected using group-aware three-fold inner cross-validation performed entirely within each outer-training partition [35]. The GCN architecture was fixed before the final repeated evaluation, and inner three-fold cross-validation was used only to determine the training duration. GCN training was limited to a maximum of 200 epochs, with early-stopping patience of 20 epochs and a minimum improvement criterion of 0.001.

For each seed, out-of-fold predictions from the five outer folds were pooled to calculate model performance. AUROC was the primary discrimination metric, with accuracy, F1 score, and Matthews correlation coefficient (MCC) reported as secondary metrics [53]. Performance was summarized as the mean ± standard deviation across the 10 seeds. Because the repeated evaluations were based on the same set of reference drugs, seed-level results were treated as repeated computational evaluations rather than independent biological observations.

As a post hoc class-held-out sensitivity analysis, cross-class transfer was evaluated by training the primary linear SVM on one antihypertensive class and testing it on the other class, which was completely excluded from training. Comparator drugs were partitioned into mutually exclusive training and test sets matched to the number of positive drugs, and 1000 unique comparator partitions were evaluated in each transfer direction. Protein vocabularies and feature scaling were derived exclusively from the training partition.

### 4.7. Prioritization of 56 Insurance-Covered Korean Herbal Formulations

Fifty-six Korean herbal formulations reimbursed under the Korean national health insurance system were evaluated. Each formulation was represented as a protein-associated PPI network using the same network-construction and preprocessing procedures applied to the reference-drug dataset.

Because the linear SVM showed the highest repeated cross-validated discrimination, it was used as the primary formulation-ranking model. Each of the 50 outer-fold SVM models independently scored the 56 formulations using only the protein vocabulary and feature-scaling parameters derived from its corresponding training partition. Formulations were prioritized according to their rank distributions across the 50 models, including median rank and top-10 frequency. The complete ranking was retained without imposing a binary therapeutic classification threshold. Secondary models were used only to assess cross-model concordance.

Sensitivity analyses were performed by re-scoring the 56 formulations with the existing 50 outer-fold SVM models without model refitting. Candidate graphs were reconstructed under harmonized STITCH combined-score thresholds, and normalized degree was recalculated within each reduced induced subgraph. Additional analyses controlled for the number of fold-recognized proteins by contribution normalization and balanced subsampling, and assessed the influence of recurrent top-10-associated proteins by ablation. Cross-disease rank concordance was examined using previously generated T2D and HLP reference models applied to the same formulation graphs.

### 4.8. Protein-Level Interpretation of the Linear SVM

Because the three SVM feature channels—protein presence, HTN disease weight, and normalized degree—represented complementary attributes of the same protein, they were interpreted jointly rather than as independent biological effects. For each protein, the contributions of the three standardized channels were therefore aggregated at the protein level.

For a held-out drug, the contribution of protein *j* to the SVM decision function was calculated as the sum of the products of its standardized feature values and corresponding SVM coefficients across the three channels:

$$C_{ijm}=\sum_{k=1}^{3}\beta_{jkm}z_{ijkm}$$

where *k* denotes the presence, HTN-weight, or normalized-degree channel, β*_jkm_* is the fitted coefficient in outer model m, and z*_ijkm_* is the corresponding standardized feature value for held-out drug *i*. Each drug contributed one out-of-fold evaluation per seed, yielding 10 repeated out-of-fold (OOF) protein-contribution estimates.

For each protein, the antihypertensive-reference-associated directional effect was defined as the difference between its mean OOF contribution in the 15 antihypertensive drugs and that in the 15 comparator drugs. A protein was included in the stable, directionally consistent SVM-derived antihypertensive-reference-associated signature if it was evaluable in at least 80% of outer models, showed a positive protein-level coefficient in at least 80% of eligible models, showed a positive HTN-versus-comparator OOF contribution difference in at least 80% of seeds, and had a positive overall OOF contribution difference.

### 4.9. Label-Permutation Calibration of the SVM Protein Signature

To assess whether the observed protein-level effects exceeded those expected under random class labeling, drug-level HTN/comparator labels were permuted 1000 times while preserving the original outer-fold assignments [54]. For each permutation, the SVM was refitted, and the protein-level OOF contribution and stability analysis was repeated. Because C = 0.1 was selected in all 50 observed inner-cross-validation fits, this value was fixed for the permutation refits.

For each observed OOF effect threshold t, the expected number of null discoveries was estimated as the mean number of proteins exceeding the same threshold across the permuted datasets. Empirical false discovery rate (FDR) was calculated as

$$FDR\left(t\right)=\frac{E[N_{null}\left(\Delta \geq t\right)]}{N_{observed}(\Delta \geq t)}$$

Empirical q-values were obtained from the threshold-specific FDR estimates using a cumulative-minimum procedure [55] to ensure monotonicity. A q-value below 0.05 was considered the strict discovery threshold, whereas q < 0.10 was regarded as exploratory. Permutation calibration was used to assess individual-protein evidence, while the specified directional-stability criteria were retained for subsequent network-level analyses.

### 4.10. Network-Module Analysis of the Stable Antihypertensive-Reference-Associated Signature

The stable SVM-derived proteins were mapped onto the PPI networks observed across the 15 antihypertensive reference drugs. An interaction was retained if it occurred in at least one antihypertensive-drug graph, and its edge weight was defined as the number of positive-drug graphs containing that interaction.

The resulting aggregated PPI network was partitioned using weighted greedy modularity maximization [56]. Community detection was performed solely on network connectivity. Descriptive functional labels were assigned post hoc based on the predominant biological characteristics of member proteins; these labels did not represent formal GO, KEGG, or other pathway-enrichment results.

For each module, the aggregate SVM signal was summarized as the mean OOF HTN-versus-comparator contribution difference of its member proteins. Statistical calibration was performed using 5000 random protein sets of the same size drawn from the 110-protein stable-signature universe. The empirical module *p*-value was calculated as

$$P_{emp}=\frac{1+\#(\Delta_{random}\geq \Delta_{observed})}{1+5000}$$

A plus-one correction was applied to avoid zero empirical *p*-values. Module size, edge count, and network density were additionally summarized for descriptive network characterization. However, these modules should not be interpreted as biologically validated functional entities or causal effector units. Rather, they represent network communities of model-associated proteins that may highlight molecular neighborhoods for subsequent mechanistic investigation.

### 4.11. Molecular Signature Coverage of Prioritized Formulations

To characterize the molecular basis of formulation prioritization, each formulation protein set was compared with the stable 110-protein SVM-derived antihypertensive-reference-associated signature. For formulation f, weighted signature coverage was defined as

$$Coverage_{f}=\frac{\sum_{j\in f\cap S}\Delta_{j}}{\sum_{j\in S}\Delta_{j}}$$

where *S* denotes the stable SVM-derived antihypertensive-reference-associated signature, and Δ*j* is the OOF HTN-versus-comparator contribution difference for protein *j*. Both the number of overlapping proteins and the weighted coverage were summarized. Module-specific coverage was additionally calculated for the M1 and M2 modules using the same approach with module-restricted denominators.

The analysis was performed across all 56 formulations to provide library-wide context. These coverage measures were used to decompose and interpret the SVM-derived molecular signal and were not considered independent external validation.

### 4.12. Blind GCN Protein-Identity Attribution

For tractability, GCN attribution was restricted to three representative high-priority formulations—Antae-eum, Hyangsapyeongwi-san, and Sigyeongbanha-tang—each of which remained within the SVM Top 10 across all 50 outer-fold models. Protein identities remained encoded during attribution and ranking and were decoded to gene symbols only after the final Top-15 attribution set had been fixed.

For each protein node, its encoded identity was replaced by the out-of-vocabulary identity while retaining its numerical node features and the original PPI connectivity [57]. The perturbed graph was then evaluated using the corresponding trained GCN. Protein-identity attribution was defined as

Δlogit = logit_*original*_ − logit_*identity occluded*_.

A positive Δlogit indicated that masking the protein identity reduced the GCN HTN prediction score. This procedure therefore measured the contribution of protein-identity information without removing the node or its network context.

Attributions were calculated across the 10 repeated-seed GCN models and summarized using mean and median Δlogit, the fraction of positive-attribution seeds, and top-20% attribution frequency. The resulting Top-15 proteins were interpreted as model-influential protein identities rather than experimentally validated pharmacological targets.

### 4.13. Cross-Model SVM–GCN Convergence and PPI Coherence

Cross-model agreement was assessed by comparing the blind GCN Top-15 attribution set with the stable SVM-derived antihypertensive-reference-associated signature. Because GCN attribution was restricted to three representative formulation networks, overlap enrichment was evaluated using the union of proteins present in these three formulations as the background universe. Enrichment of the observed overlap was tested using the exact upper-tail hypergeometric distribution [58]. Spearman correlation was additionally used to compare GCN attribution magnitude with the corresponding SVM OOF contribution values.

Network coherence of the GCN Top-15 proteins was evaluated within the same candidate-protein universe. The number of PPI edges among the 15 attributed proteins was compared with 10,000 random sets of 15 proteins drawn from that universe, and an empirical *p*-value was calculated using the plus-one correction.

Because the SVM and GCN were derived from overlapping molecular input data, these analyses were interpreted as internal cross-model mechanistic agreement rather than independent experimental validation.

### 4.14. External Comparison with Independently Measured Vasorelaxant Activity

To evaluate the formulation ranking against measurements generated outside the present framework, it was compared with a previously published screen in which the vasorelaxant activity of 50 commonly used traditional herbal prescriptions was measured on isolated rat aortic rings pre-contracted with phenylephrine [21]. Twenty-two of the 56 formulations evaluated here were present in that panel. The mean SVM decision score of each formulation across the 50 outer-fold models was compared with the reported relaxation at 1000 µg/mL using Spearman rank correlation, with significance additionally assessed by 20,000 random permutations of the measured values and robustness by leave-one-out resampling.

### 4.15. Statistical Analysis, Reproducibility, and Interpretation

Primary classification performance was assessed using AUROC, with accuracy, F1 score, and Matthews correlation coefficient (MCC) reported as secondary metrics. Performance was summarized as the mean ± standard deviation across the 10 repeated seeds. Because repeated cross-validation reused the same 30 reference-drug graphs, seed-level results were treated as repeated computational evaluations rather than statistically independent observations.

For uncertainty estimation at the independent-drug level, the 10 repeated OOF SVM decision scores for each reference drug were averaged, and AUROC was recalculated using the resulting 30 drug-level scores. A stratified 95% bootstrap confidence interval was obtained by independently resampling the 15 antihypertensive and 15 comparator drugs with replacement over 10,000 replicates.

Empirical randomization procedures were used for SVM protein-level calibration, module-level aggregate effects, and GCN-attributed PPI coherence, as described above. Exact upper-tail hypergeometric testing was used to evaluate SVM–GCN protein-set overlap, and Spearman rank correlation was used for rank-based cross-model comparisons. Statistical results were interpreted in the context of the exploratory design and the limited size of the reference-drug dataset.

All analyses and numerical outputs were generated programmatically from the study datasets. Reproducibility records included cross-validation assignments, model configurations, out-of-fold predictions, formulation rankings, protein-level contribution statistics, permutation null distributions, module assignments, GCN attribution outputs, and source data used for manuscript figures and tables.

Analyses were conducted in Python 3.13.5 using PyTorch 2.10.0 (CPU build) [59], scikit-learn 1.8.0 [60], pandas 2.2.3, NumPy 2.3.5 [61], SciPy 1.17.0, NetworkX 3.6.1 [62], matplotlib 3.10.8, and openpyxl 3.1.5.

## 5. Conclusions

This study established a hypertension-focused computational framework for prioritizing insurance-covered Korean herbal formulations using molecular patterns learned from marketed antihypertensive drug networks. Accordingly, highly ranked formulations should not be interpreted as formulations predicted to lower blood pressure, because the present framework does not distinguish agonistic from antagonistic or other directionally opposing pharmacological actions. Rather, the rankings provide a drug-referenced molecular prioritization for subsequent mechanistic and experimental evaluation. The linear SVM provided the highest predictive performance, while protein- and network-level analyses revealed complementary ion-channel and receptor-associated components of the antihypertensive-reference-associated molecular signature. Prioritized formulations showed their greatest stable-signature overlap within the receptor-associated M1 network, whereas M2 proteins were sparsely represented across the formulation networks. These findings provide a reproducible basis for experimental prioritization and mechanistic hypothesis generation, but should not be interpreted as evidence of clinical antihypertensive efficacy.

## Figures and Tables

**Figure 1:**
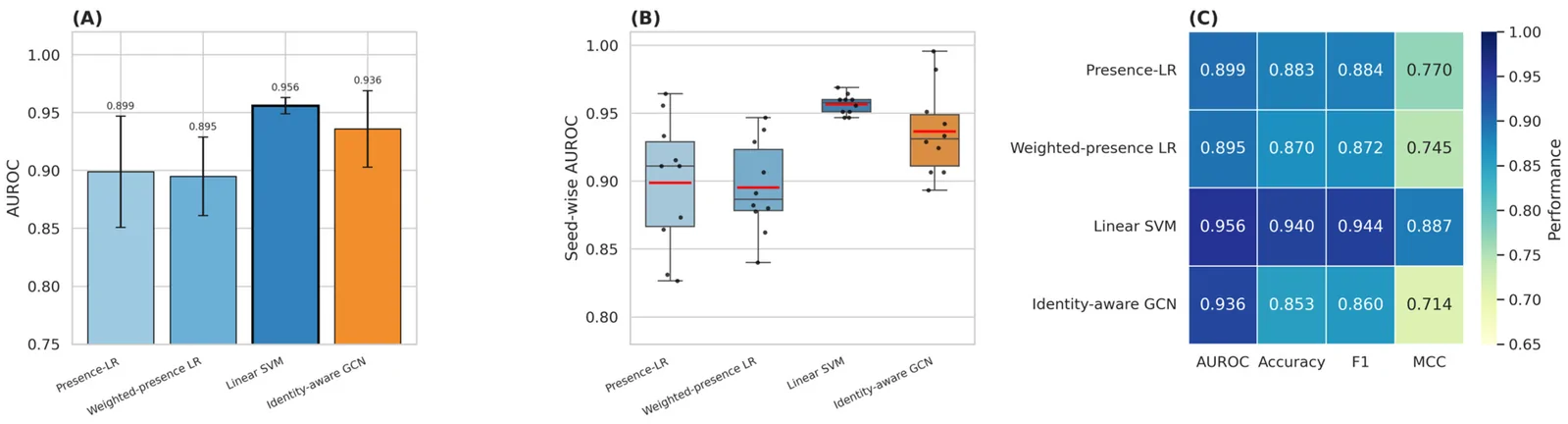
Comparative performance of four classifiers for hypertension drug discrimination. (**A**) Mean AUROC ± SD across 10 repeated group-aware outer-validation seeds. (**B**) Seed-wise AUROC distributions for each model. Red horizontal lines indicate mean AUROC values. (**C**) Heatmap summarizing mean AUROC, accuracy, F1 score, and Matthews correlation coefficient (MCC). Presence-LR, elastic-net logistic regression using binary protein presence; Weighted-presence LR, elastic-net logistic regression using hypertension-weighted protein presence; Linear SVM, linear support vector machine using protein identity, hypertension disease weight, and normalized degree; Identity-aware GCN, graph convolutional network using protein identity, hypertension disease weight, normalized degree, and full PPI edges.

**Figure 2:**
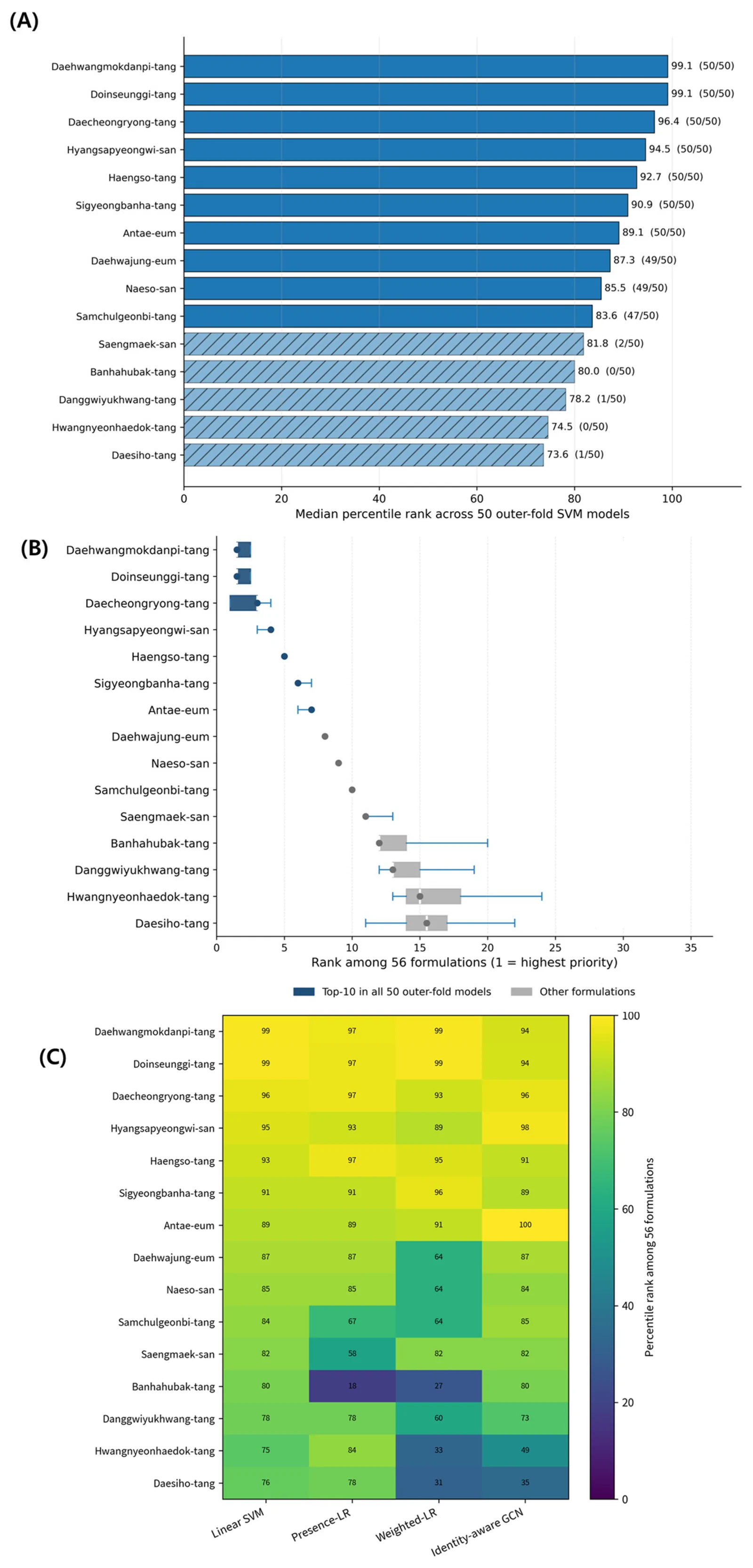
SVM-based prioritization and cross-model comparison of 56 insurance-covered Korean herbal formulations. (**A**) Top 15 herbal formulations according to the primary SVM rank-stability ranking. Bars represent the median percentile rank across 50 outer-fold SVM models, with higher values indicating more consistently favorable rankings. Numbers to the right of each bar indicate the median percentile rank followed by the number of models in which the formulation ranked within the top 10 (Top-10 count/50). Solid bars indicate the overall top 10 formulations, whereas hatched bars indicate formulations ranked 11th–15th. (**B**) Rank stability of the top 15 formulation candidates across 50 outer-fold linear SVM models. Boxplots show the distribution of formulation ranks among the 56 insurance-covered formulations; lower ranks indicate higher priority. Formulations appearing in the Top 10 in all 50 models are shown in dark blue, whereas the remaining formulations are shown in gray. Dots indicate median ranks, and the labels on the right indicate Top-10 frequency. (**C**) Cross-model comparison of the leading SVM-ranked formulations using percentile-based rankings from Presence-LR, Weighted-presence LR, linear SVM, and identity-aware GCN models. Higher percentile values indicate higher relative priority within the 56-formulation library. Rankings represent computational prioritization rather than predicted clinical efficacy.

**Figure 3:**
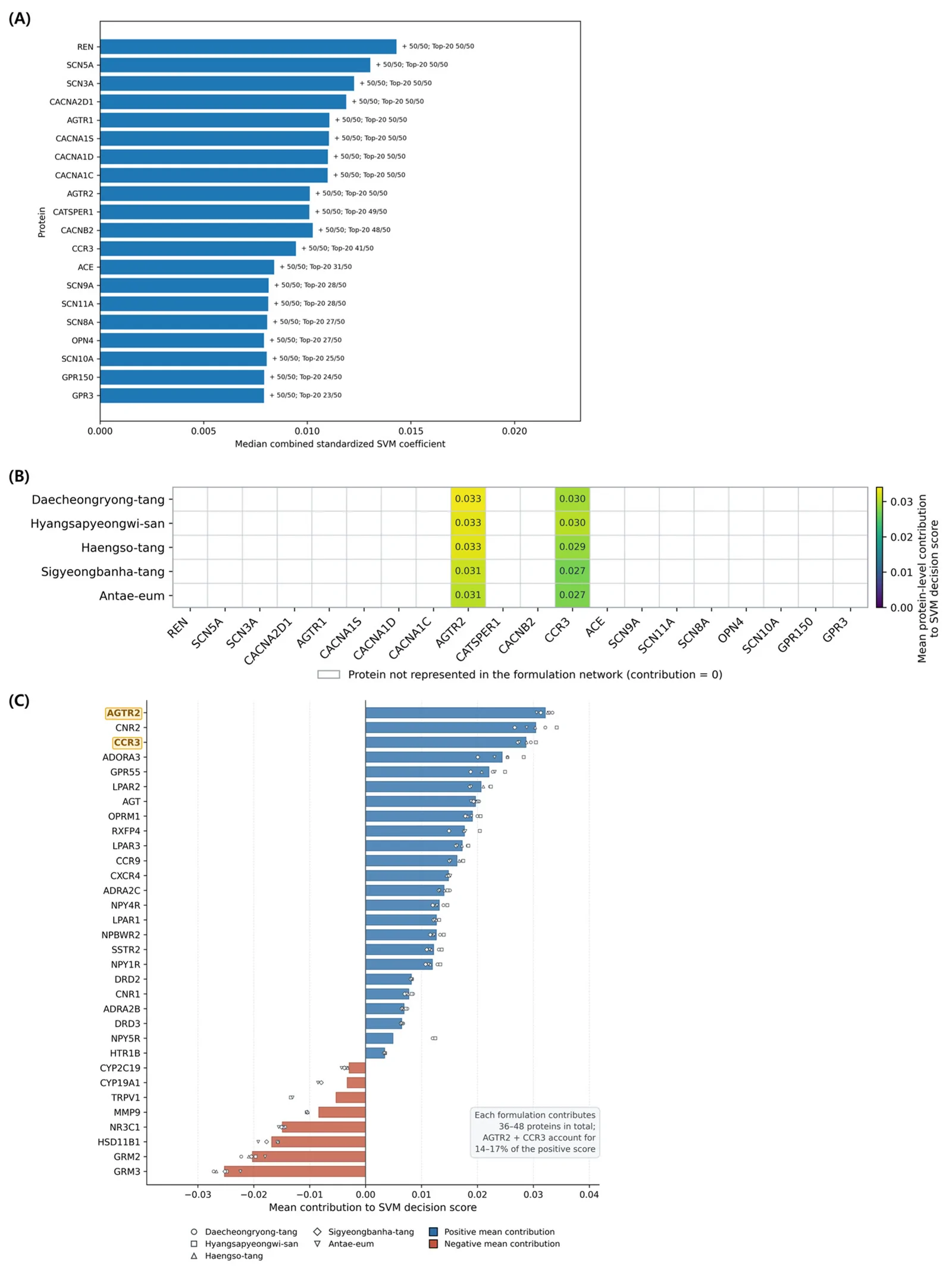
Protein-level interpretation of the linear SVM and decomposition of formulation scores. (**A**) Top 20 recurrent positive protein signals ranked by the median protein-level SVM coefficient across repeated outer-fold models. Protein-level coefficients combine the three standardized SVM feature channels: protein presence, HTN-specific disease weight, and normalized degree. (**B**) Protein-level contributions of the (**A**) Top-20 proteins to the SVM decision score for representative high-priority formulations. White cells indicate proteins not represented in the formulation network, for which the contribution is exactly zero. Colored cells indicate proteins present in the network, with the value giving the mean contribution across the 50 outer-fold models. (**C**) Protein-level contributions to the SVM decision score across five representative high-priority formulations. Bars give the mean contribution across the five formulations; open symbols give the value for each individual formulation. Blue and orange denote positive and negative mean contributions, respectively. Proteins with a mean absolute contribution above 0.002 are shown. AGTR2 and CCR3 (highlighted labels) are the only two proteins shared with the Top-20 drug-discriminative set in panel (**A**).

**Figure 4:**
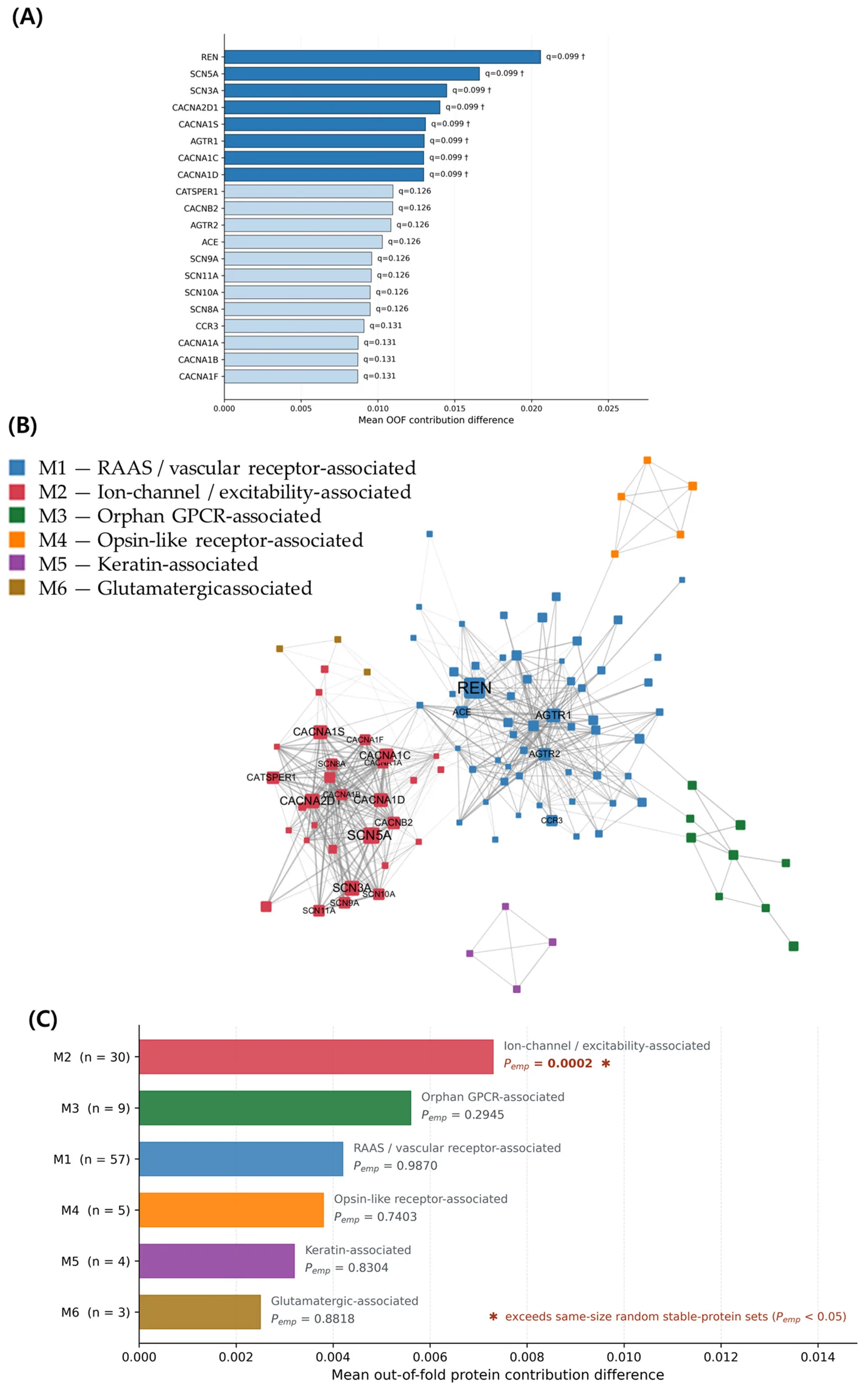
Protein-level SVM interpretation and PPI module analysis of the antihypertensive-reference-associated signature. (**A**) Top stable antihypertensive reference-associated proteins ranked by mean out-of-fold (OOF) contribution difference between antihypertensive drugs and comparator drugs. Dark bars indicate exploratory empirical q < 0.10; light bars indicate q ≥ 0.10. No protein reached empirical q < 0.05. † exploratory empirical q < 0.10. (**B**) PPI network of the stable SVM-derived antihypertensive-reference-associated signature. Node colors denote modules identified by weighted greedy modularity maximization, and node size is proportional to the mean out-of-fold (OOF) HTN-versus-comparator contribution difference. Gene symbols are displayed for the Top 20 proteins ranked by mean OOF contribution difference. Edge width is proportional to edge weight, defined as the number of antihypertensive reference-drug graphs in which the corresponding interaction was observed. (**C**) Aggregate effect size of the six PPI modules. Bars give the mean out-of-fold protein contribution difference for the proteins assigned to each module; module colors match panel (**B**). Empirical *p* values were obtained by comparison with 10,000 same-size random sets drawn from the stable signature. Only M2 exceeded the random-set distribution.

**Figure 5:**
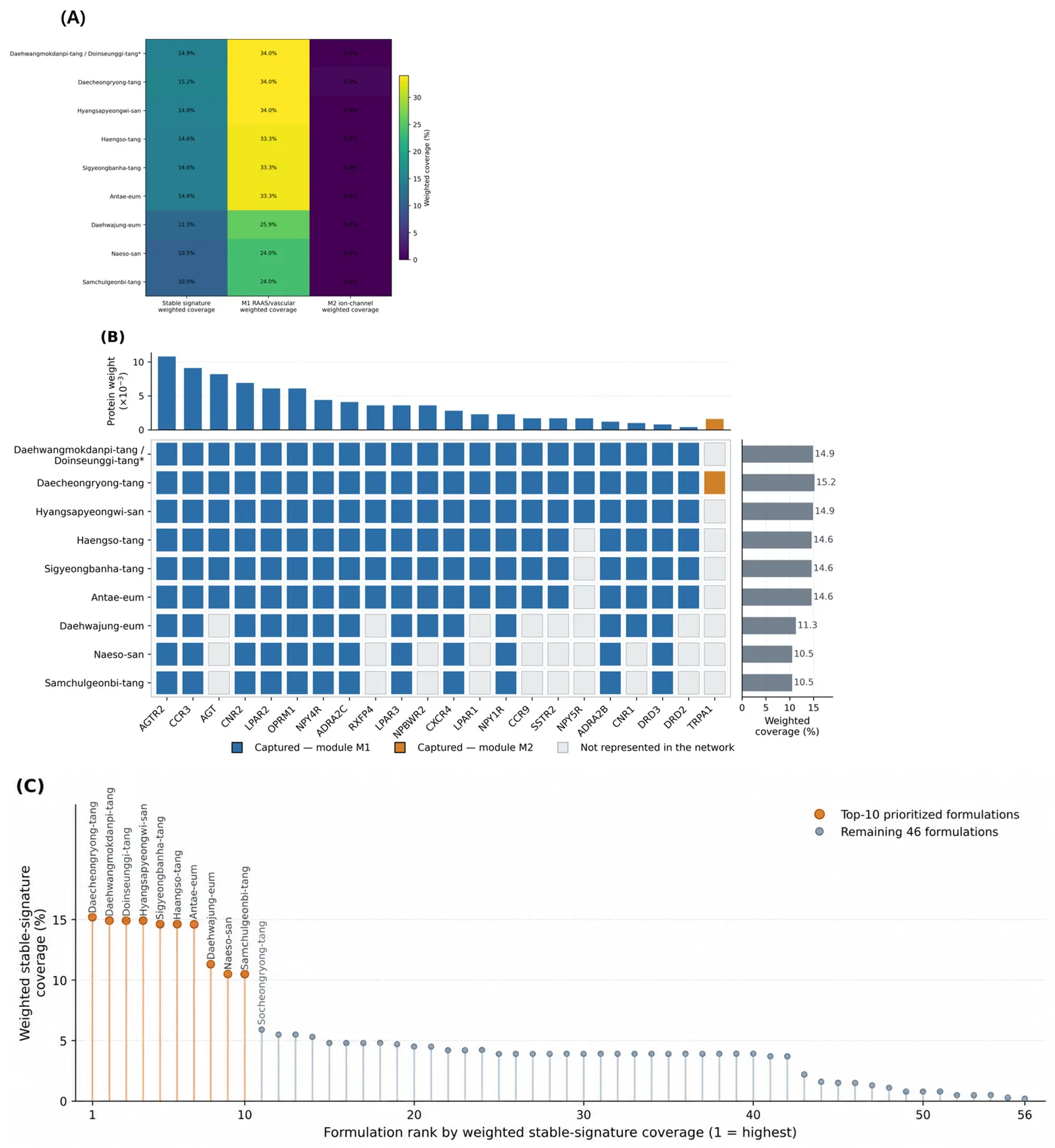
Coverage of the stable hypertension protein signature by the prioritized herbal formulations. (**A**) Weighted coverage of the full stable signature, the M1 RAAS/vascular module, and the M2 ion-channel module for the leading formulations. Each protein is weighted by its mean out-of-fold contribution difference. Asterisk denotes two formulations with identical protein sets displayed as a single row. (**B**) Stable-signature proteins captured by the top candidate networks. The upper panel gives the weight of each protein (mean out-of-fold contribution difference); proteins are ordered by weight. In the matrix, filled squares indicate proteins present in the formulation network, coloured by PPI module assignment (blue, M1; orange, M2), and open gray squares indicate proteins not represented. The right panel gives the weighted coverage of the stable signature for each formulation. The asterisk denotes two formulations with identical protein sets displayed as a single row. (**C**) Library-wide context of stable-signature coverage. All 56 insurance-covered formulations are ordered by weighted stable-signature coverage. Orange denotes the ten prioritized formulations and grey the remainder.

**Figure 6:**
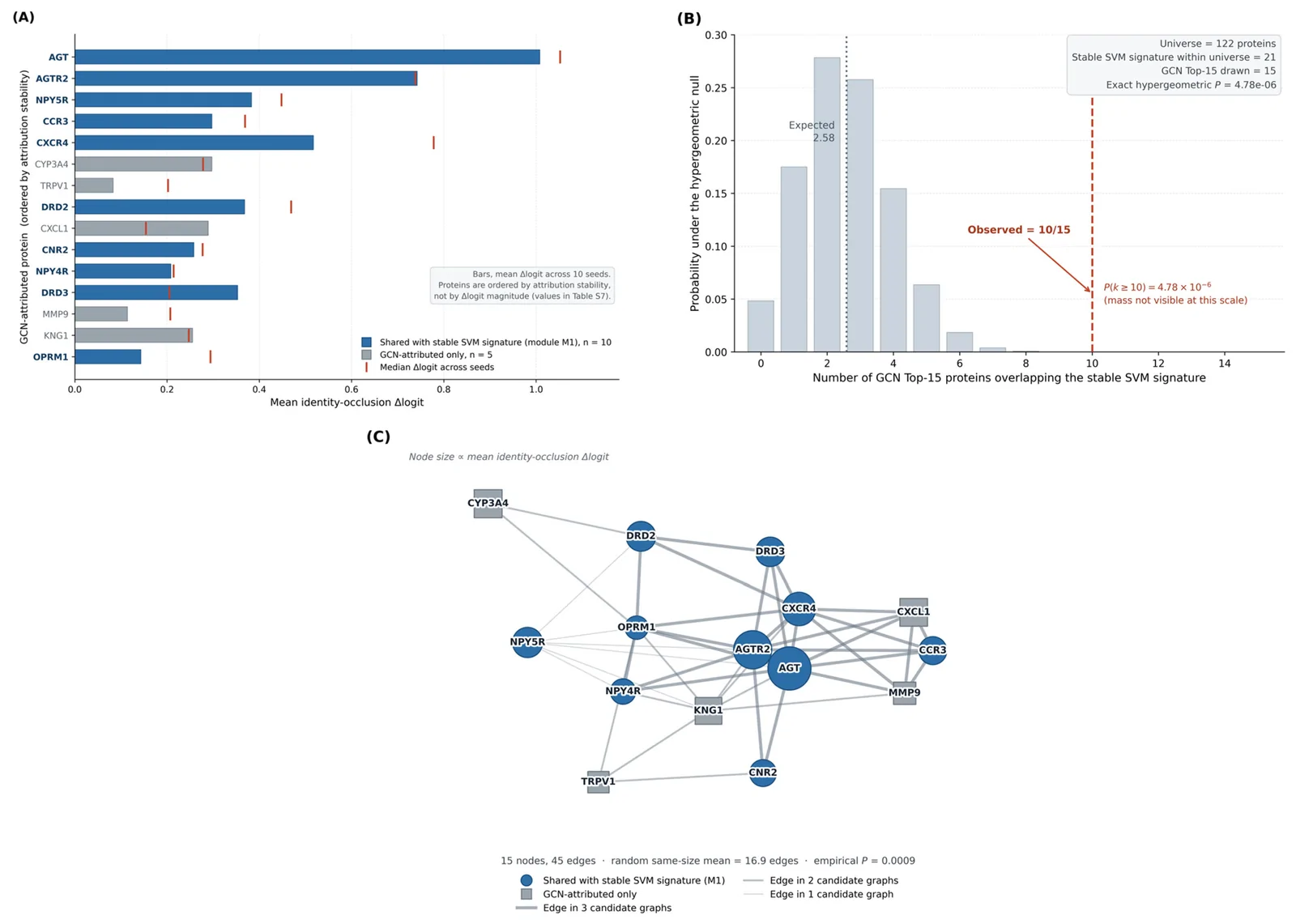
Blind GCN protein-identity attribution and cross-model comparison with the stable SVM signature. (**A**) Blind GCN protein-identity attribution in three representative high-priority formulations. Bars give the mean identity-occlusion Δlogit across 10 seeds, and red vertical marks the median. Proteins are ordered by attribution stability (top-20% seed fraction, then positive seed fraction), not by Δlogit magnitude; the corresponding values are annotated to the right of each bar. Blue denotes proteins shared with the stable SVM signature (all assigned to module M1) and grey denotes proteins attributed by the GCN only. (**B**) Cross-model overlap relative to the candidate-protein universe. The histogram gives the hypergeometric null distribution for the number of GCN Top-15 proteins overlapping the stable SVM signature, drawn from the 122-protein union of the three representative formulation networks, of which 21 belong to the stable signature. The dotted line marks the expected overlap and the dashed line the observed overlap. (**C**) GCN-attributed protein subnetwork across the three representative candidate graphs. Node color and shape denote proteins shared with the stable SVM signature (blue circles, all module M1) or attributed by the GCN only (gray squares); node size is proportional to the mean identity-occlusion Δlogit. Edge width is proportional to the number of candidate graphs (1–3) in which the interaction was observed.

**Figure 7:**
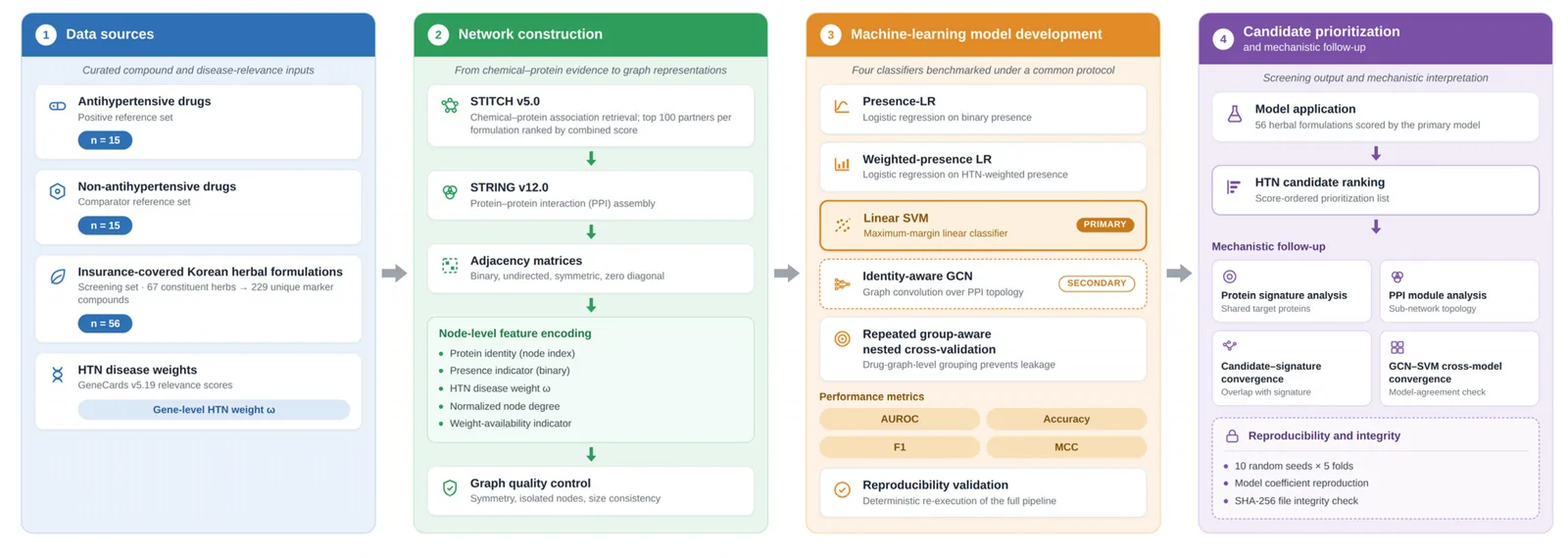
HTN-centered overall workflow for disease-specific model development and herbal formulation prioritization.

**Table 1:** Comparative predictive performance of HTN classifiers.

Model	Input Representation	AUROC Mean ± SD	Accuracy Mean ± SD	F1 Mean ± SD	MCC Mean ± SD
Linear SVM	Protein-specific presence + HTN disease weight + normalized degree	0.956 ± 0.007	0.940 ± 0.014	0.944 ± 0.013	0.887 ± 0.026
Identity-aware GCN	Protein identity + HTN disease weight + normalized degree + actual PPI edges	0.936 ± 0.033	0.853 ± 0.063	0.860 ± 0.058	0.714 ± 0.120
Presence-LR	Binary protein presence	0.899 ± 0.048	0.883 ± 0.055	0.884 ± 0.056	0.770 ± 0.109
Weighted-presence LR	Protein presence × HTN disease weight	0.895 ± 0.034	0.870 ± 0.055	0.872 ± 0.054	0.745 ± 0.110

**Table 2:** Network organization of the stable SVM-derived antihypertensive-reference-associated protein signature.

Module	Descriptive Biological Label	Proteins (*n*)	Edges (*n*)	Density	Mean OOF Contribution Difference	Empirical P (Random Same-Size Sets)	Representative Proteins
M1	RAAS/vascular receptor-associated	57	269	0.169	0.0042	0.9870	REN; AGTR1; AGTR2; ACE; CCR3; AGT; EDNRB; CNR2; OPN4; FFAR4; LTB4R2; LPAR2
M2	Ion-channel/excitability-associated	30	235	0.540	0.0073	0.0002	SCN5A; SCN3A; CACNA2D1; CACNA1S; CACNA1C; CACNA1D; CATSPER1; CACNB2; SCN9A; SCN11A; SCN10A; SCN8A
M3	Orphan GPCR-associated	9	11	0.306	0.0056	0.2945	GPR3; GPR12; GPR150; GPR61; GPR176; GPR135; GPR139; GPR78; GPR52
M4	Opsin-like receptor-associated	5	10	1.000	0.0038	0.7403	OPN5; OPN1LW; OPN1MW; RGR; OPN3
M5	Keratin-associated	4	6	1.000	0.0032	0.8304	KRT71; KRT74; KRT72; KRT73
M6	Glutamatergic-associated	3	3	1.000	0.0025	0.8818	GRIA4; GAD2; GRIA3

Of the 110 stable proteins, 108 were assigned to the six PPI communities; ADRB3 and ABCB11 were isolated in the induced subnetwork and therefore remained unassigned.

**Table 3:** Top-ranked herbal formulation networks and convergence with the stable antihypertensive-reference-associated signature.

Formulation Network	Median SVM Rank	Top-10 Frequency (*n*/50)	Network Nodes	Stable Signature Protein Captured (*n*)	Weighted Stable-Signature Coverage (%)	M1 RAAS/Vascular Coverage (%)	M2 Proteins Captured (*n*)
Daehwangmokdanpi-tang, Doinseunggi-tang	1	50	100	21	14.9	34.0	0
Daecheongryong-tang	3	50	100	22	15.2	34.0	1
Hyangsapyeongwi-san	4	50	99	21	14.9	34.0	0
Haengso-tang	5	50	100	20	14.6	33.3	0
Sigyeongbanha-tang	6	50	100	20	14.6	33.3	0
Antae-eum	7	50	98	20	14.6	33.3	0
Daehwajung-eum	8	49	98	14	11.3	25.9	0
Naeso-san	9	49	97	12	10.5	24.0	0
Samchulgeonbi-tang	10	47	98	12	10.5	24.0	0

**Table 4:** Cross-model mechanistic convergence between GCN attribution and the stable SVM antihypertensive-reference-associated network.

Analysis	Observed Result	Null/Comparator	Interpretation
GCN blind attribution	15 proteins	Top 15 ranked by identity-occlusion attribution across three representative HTN candidates	Model-influential protein identities; not causal therapeutic targets
Overlap with stable SVM signature	10/15 proteins	Expected overlap = 2.58	Hypergeometric *p* = 4.78 × 10^−6^
Shared SVM–GCN proteins	AGT, AGTR2, NPY5R, CCR3, CXCR4, DRD2, CNR2, NPY4R, DRD3, OPRM1	All 10 shared proteins mapped to SVM Module 1	Architecture-level convergence on the RAAS/vascular receptor-associated candidate module
GCN-attributed-only proteins	CYP3A4, TRPV1, CXCL1, MMP9, KNG1	Not members of the 110-protein stable SVM signature	Complementary GCN-sensitive candidate-network components
Effect-rank concordance	Spearman rho = 0.364	*p* = 0.182	Protein rank magnitudes were only modestly concordant
PPI coherence of GCN Top 15	45 induced edges	Random 15-protein mean = 16.94 edges	10,000-set empirical *p* = 0.0009
Candidate-protein universe	122	21 stable-SVM proteins in universe	Union of Antae-eum, Hyangsapyeongwi-san, and Sigyeongbanha-tang graphs

GCN attribution is formulation-centric. These analyses demonstrate internal cross-model mechanistic convergence within representative candidate graphs, not independent disease-wide validation.

**Table 5:** HTN benchmark dataset and graph characteristics.

Characteristic	Value	Definition/Note
HTN-positive drug graphs	15	Final positive reference set
Comparator drug graphs	15	Balanced negative comparator set
Total benchmark graphs	30	15 positive + 15 comparator
Repeated seeds	10	11, 22, 33, 42, 55, 66, 77, 88, 99, 123
Outer folds	5	Group-aware stratified outer CV
HTN protein vocabulary	1254	Distinct encoded proteins in the final HTN benchmark vocabulary
Total nodes across 30 graphs	2257	Sum across final benchmark graphs
Total edges across 30 graphs	14,430	Sum across final benchmark graphs
Median nodes per graph	77.5	Median across 30 graphs
Median edges per graph	346	Median across 30 graphs
Mean HTN-weight mapping ratio	46.2%	Fraction of graph proteins with mapped HTN disease weights

## Data Availability

All data and code required to reproduce the reported results are openly available. The complete reproducibility package—including the curated reference-drug and formulation network datasets, cross-validation fold assignments, model configurations, analysis code, out-of-fold predictions, permutation null distributions, and the source data underlying every figure and table—has been deposited in Zenodo at https://doi.org/10.5281/zenodo.22654771.

## Supplementary Materials

The following supporting information can be downloaded at: https://www.mdpi.com/article/10.3390/ijms27198548/s1.

## Abbreviations

The following abbreviations are used in this manuscript:

AUROC	Area under the receiver operating characteristic curve
CPU	Central processing unit
FDR	False discovery rate
GCN	Graph convolutional network
GPCR	G protein-coupled receptor
HIRA	Health Insurance Review & Assessment Service
HTN	Hypertension
KFDA	Korea Food and Drug Administration
LR	Logistic regression
M1–M6	Protein–protein interaction network modules 1–6
MCC	Matthews correlation coefficient
OOF	Out-of-fold
PPI	Protein–protein interaction
RAAS	Renin–angiotensin–aldosterone system
SD	Standard deviation
STITCH	Search Tool for Interactions of Chemicals
STRING	Search Tool for the Retrieval of Interacting Genes/Proteins
SVM	Support vector machine
