# Supplementary material for: A Drug-Referenced Machine-Learning Framework for Prioritizing Korean Herbal Formulations in Hypertension Using Linear SVM and Graph Convolutional Networks
Source: Int J Mol Sci. 2026 Sep 24;27(19):8548. doi: 10.3390/ijms27198548 (PMC13665111; doi:10.3390/ijms27198548)
Supplement: Supplementary file 1 [file ijms-27-08548-s001.zip › ijms-4534525-supplementary.pdf]

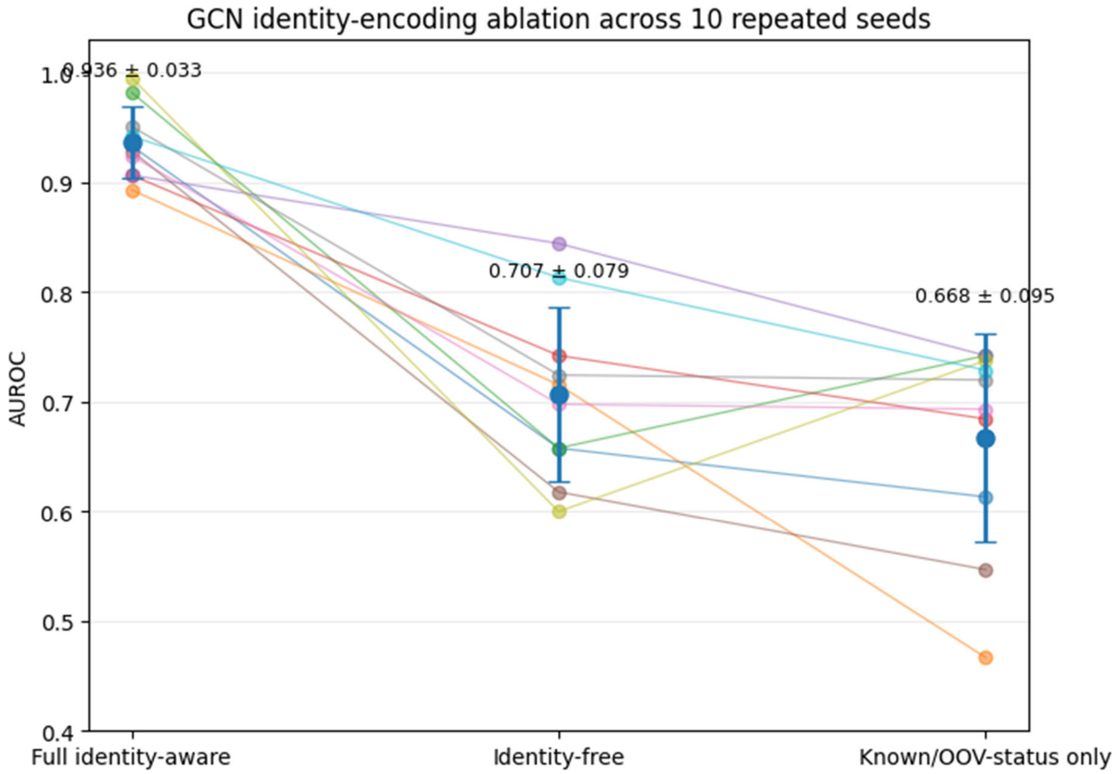

Figure S1 Ablation of protein-identity encoding in the identity-aware GCN across 10 repeated seeds.

Out-of-fold AUROC is shown for three input configurations evaluated under identical seed-specific outer-fold assignments and the training epochs selected in the primary repeated-seed validation. Full identity-aware, the primary GCN configuration combining a learnable per-protein identity embedding with the four numerical node features and the binary PPI adjacency; Identity-free, the identity embedding removed, retaining only the numerical node features and adjacency; Known/OOV-status only, the per-protein identity replaced by a single shared learnable vector indicating whether a protein was present in the outer-training vocabulary. Thin lines connect the same seed across configurations, small points give seed-level values, and large points with error bars give the mean ± standard deviation across the 10 seeds, annotated above each configuration.

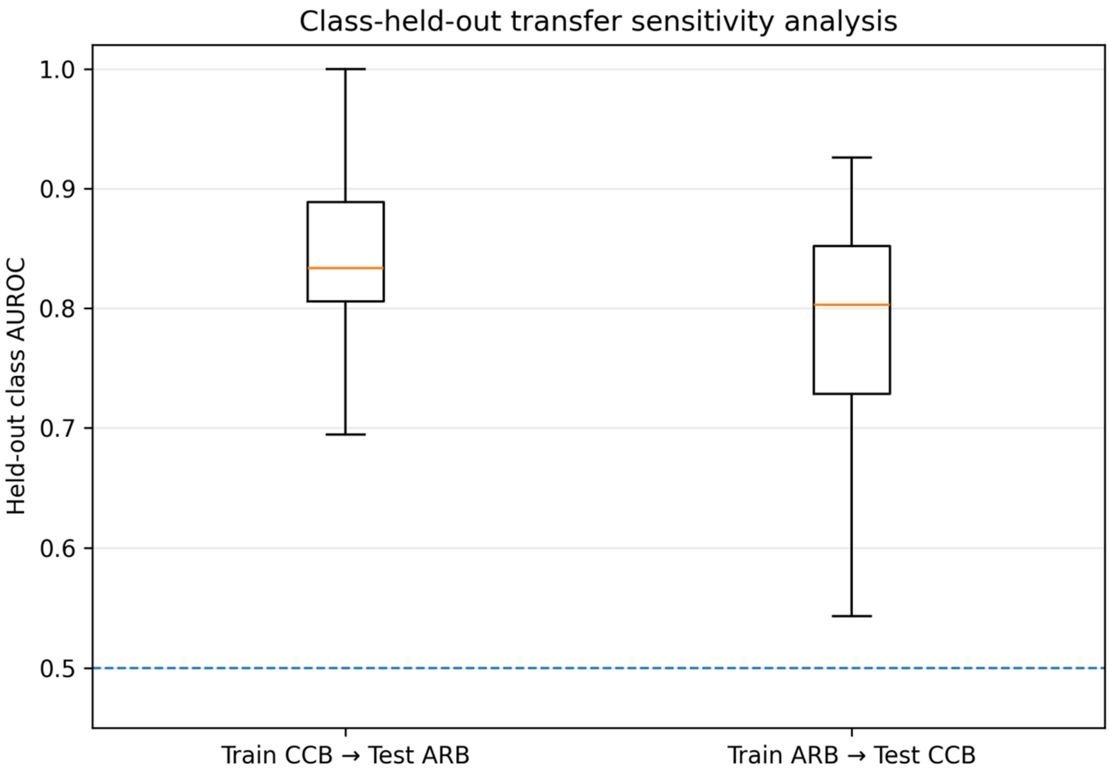

Figure S2 Class-held-out transfer of the linear SVM between the two antihypertensive drug classes.

Boxplots show held-out-class AUROC across 1,000 exhaustive comparator partitions. In the left condition the model was trained on the nine calcium-channel blockers with nine comparator drugs and evaluated on the six angiotensin receptor blockers with the six held-out comparators; in the right condition the directions were reversed. Boxes give the interquartile range and orange lines the median; whiskers extend to the most extreme values within 1.5 times the interquartile range.

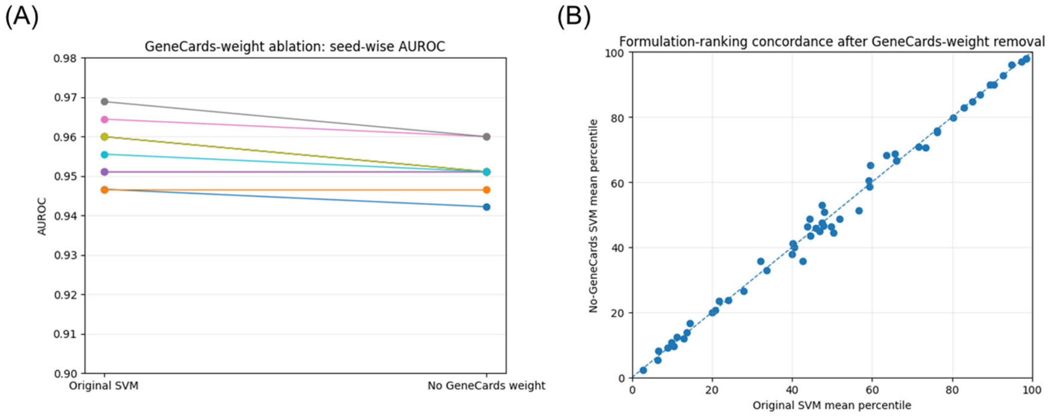

(C)

Analysis	Original SVM	No-GeneCards SVM
AUROC	0.956 ± 0.007	0.952 ± 0.005
Accuracy	0.940 ± 0.014	0.937 ± 0.011
F1	0.944 ± 0.013	0.941 ± 0.010
MCC	0.887 ± 0.026	0.881 ± 0.019
Stable proteins	110	108

Figure S3 Ablation of the hypertension-specific GeneCards disease-weight channel from the primary linear SVM.

The SVM was refitted using only two of the three protein-specific feature channels — binary protein presence and normalized graph degree — under the same seed-specific outer-fold assignments, training-partition vocabularies and feature scaling as the primary model.

(A) Seed-wise out-of-fold AUROC for the original three-channel SVM and the two-channel model without the GeneCards weight. Each line connects the same seed across the two configurations; overlapping lines indicate seeds with identical values.

(B) Concordance of the formulation ranking before and after removal of the disease-weight channel. Each point is one of the 56 insurance-covered Korean herbal formulations, plotted by its mean percentile rank across the 50 outer-fold models under each configuration. The dashed line indicates identity.

(C) Discrimination metrics summarized as mean ± standard deviation across the 10 repeated seeds, together with the size of the directionally stable protein signature obtained under each configuration.

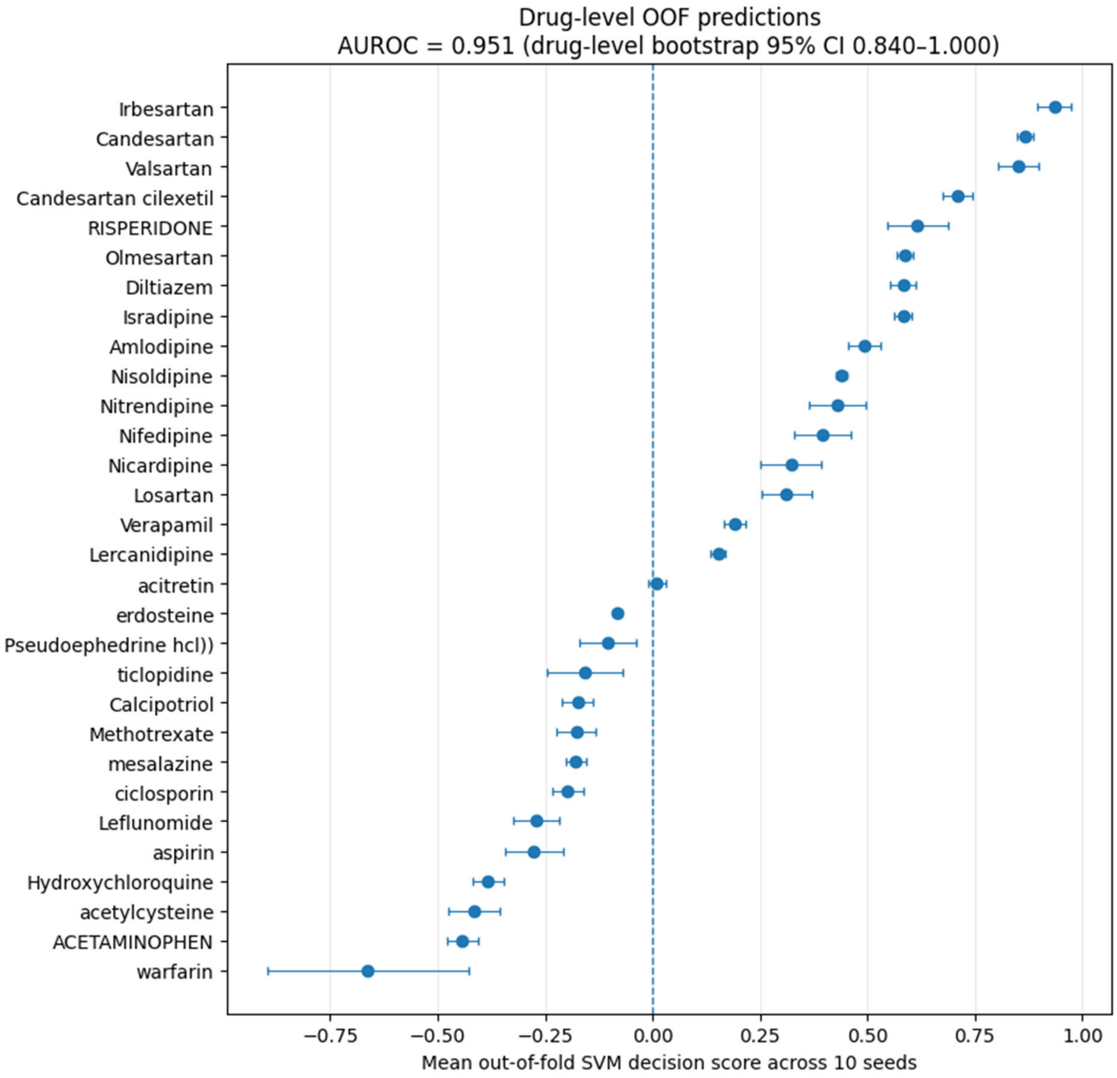

- **Figure S4** Drug-level out-of-fold predictions of the primary linear SVM for the 30 reference drugs.

  Points give the mean out-of-fold SVM decision score for each drug across the 10 repeated seeds, and horizontal bars give ± 1 standard deviation across those seeds. Drugs are ordered by mean decision score. The dashed vertical line marks the decision boundary at zero; positive values indicate assignment toward the antihypertensive reference class. The AUROC shown in the title was calculated on these drug-level mean scores, with a 95% confidence interval obtained by bootstrap resampling of the 30 drugs (10,000 resamples).

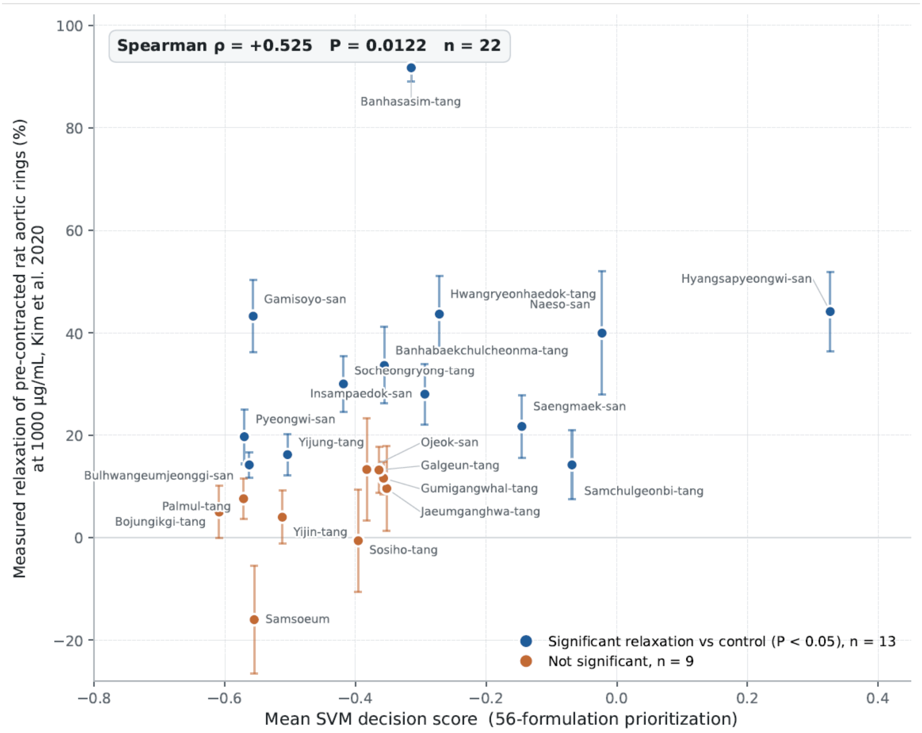

Figure S5. Comparison of the formulation ranking with independently measured vasorelaxant activity.

Each point is one of the 22 formulations common to the present library and a published screen of 50 traditional herbal prescriptions on isolated rat aortic rings [1]. The horizontal axis gives the mean SVM decision score across the 50 outer-fold models; the vertical axis gives the reported relaxation of phenylephrine-pre-contracted aortic rings at 1000 μg/mL, with error bars showing the standard error of the mean. Blue denotes formulations reported as producing significant relaxation relative to control and orange those that did not; negative values indicate net contraction.

Table S1. Network characteristics of the 30 reference drugs comprising the hypertension benchmark dataset.

Reference class	Drug	Category/Indication	Network nodes	PPI edges
**Antihypertensive**	Amlodipine	CCB	76	366
	Candesartan cilexetil*	ARB	69	198
	Candesartan	ARB	79	352
	Diltiazem	CCB	68	251
	Irbesartan	ARB	80	346
	Isradipine	CCB	59	300
	Lercanidipine	CCB	44	111
	Losartan	ARB	76	826
	Nicardipine	CCB	77	275
	Nifedipine	CCB	87	552
	Nisoldipine	CCB	60	185
	Nitrendipine	CCB	81	430
	Olmesartan	ARB	81	306
	Valsartan	ARB	82	337
	Verapamil	CCB	91	469
**Comparator**	Acetaminophen	Analgesic–antipyretic	92	793
	Calcipotriol	Vitamin D analogue (topical)	38	129
	Hydroxychloroquine	Antimalarial/ DMARD	69	267
	Leflunomide	Disease-modifying antirheumatic drug (DMARD)	82	360
	Methotrexate	Disease-modifying antirheumatic drug (DMARD)	70	286
	Pseudoephedrine	Sympathomimetic decongestant	69	260
	Risperidone	Atypical antipsychotic	78	437
	Acetylcysteine	Mucolytic	90	1247
	Acitretin	Systemic retinoid	71	336
	Aspirin	Antiplatelet /	94	1538

	NSAID		
Ciclosporin	Calcineurin inhibitor/ immunosuppressant	99	1143
Erdosteine	Mucolytic	53	225
Mesalazine	5-Aminosalicylate (5-ASA) / anti-inflammatory agent	74	346
Ticlopidine	Antiplatelet	79	887
Warfarin	Anticoagulant	89	872

*Candesartan cilexetil is the ester prodrug of candesartan; both were retained as distinct STITCH chemical records with partially distinct protein annotation. Their target-set overlap (Jaccard 0.54) was lower than that of other angiotensin-receptor-blocker pairs such as candesartan–valsartan (0.68), and mean within-class overlap was 0.38 for angiotensin receptor blockers versus 0.04 between classes.

Table S2. Seed-wise reproducibility and coefficient-reproduction quality control of the primary linear SVM. (A) Out-of-fold (OOF) discrimination for each of the 10 predetermined random seeds, obtained by re-executing the exact primary SVM pipeline. AUROC, area under the receiver operating characteristic curve; F1, F1 score; MCC, Matthews correlation coefficient. (B) Quality-control summary of coefficient reproduction and permutation calibration. The re-executed pipeline reproduced all 160,221 stored channel coefficients (maximum absolute difference 1.38×10^{-10}; mean 3.53×10^{-12}) and all 10 seed-wise AUROC values. Permutation calibration used 1,000 label-permutation replicates; 110 proteins satisfied the predefined directional-stability criteria, none reached the strict threshold of empirical $q < 0.05$, and eight reached the exploratory threshold of $q < 0.10$.

**A. Seed-wise out-of-fold performance reproduced from the exact primary SVM pipeline**				
**Seed**	**AUROC**	**Accuracy**	**F1**	**MCC**
11	0.947	0.967	0.968	0.935
22	0.947	0.933	0.938	0.874
33	0.960	0.933	0.938	0.874
42	0.951	0.933	0.938	0.874
55	0.951	0.933	0.938	0.874
66	0.960	0.933	0.938	0.874
77	0.964	0.933	0.938	0.874
88	0.969	0.967	0.968	0.935
99	0.960	0.933	0.938	0.874
123	0.956	0.933	0.938	0.874

**B. Coefficient reproduction QC**	
**QC metric**	**Value**
Compared channel coefficients	160221
Maximum absolute difference	1.38E-10
Mean absolute difference	3.53E-12
99th percentile absolute difference	4.53E-11
Permutation replicates	1000
Stable directional proteins	110
Proteins with empirical q < 0.05	0
Proteins with empirical q < 0.10	8

The exact SVM pipeline reproduced 160,221 stored channel coefficients with maximum absolute difference 1.38×10^-10 and reproduced all 10 seed-wise AUROCs.

Table S3. Sensitivity analyses of the formulation ranking under harmonized candidate-graph construction and graph-size controls. All analyses re-scored the 56 formulations using the existing 50 outer-fold SVM models without refitting; model coefficients, disease weights, and outer-fold assignments were held fixed. Panel S-a reconstructed candidate graphs under harmonized STITCH combined-score floors, either with variable graph size (uniform floor only) or with a fixed graph size (balanced design). Panel S-b controlled for the number of proteins represented in the fold-specific training vocabulary, by re-ranking on mean contribution per recognized protein and by balanced subsampling to a common number of recognized proteins (200 iterations). Panel S-c removed the 18 training-vocabulary proteins shared across the Top-10 formulations, with removal of an equal number of randomly selected vocabulary proteins (20 iterations) as a control. Spearman ρ is computed against the original median formulation ranking; Top-10 retained indicates how many of the original Top-10 formulations remained within the Top 10. Normalized degree was recomputed as degree/(n − 1) within each reduced induced subgraph.

Panel	Analysis	Design	Spearman rho vs original	Top-10 retained	Nodes (min–median–max)
S-a	Confidence-threshold reconstruction	Uniform STITCH floor c=0.300 (variable size)	1	10 / 10	73–97–100
S-a	Confidence-threshold reconstruction	Uniform STITCH floor c=0.400	1	10 / 10	51–97–100
S-a	Confidence-threshold reconstruction	Uniform STITCH floor c=0.700	0.997	10 / 10	19–96–100
S-a	Confidence-threshold reconstruction	Balanced: floor c=0.300 + fixed size k=78	0.876	10 / 10	68–78–78
S-b	Graph-size / vocabulary controls	Re-rank by mean contribution per recognized protein	0.956	10 / 10	n/a
S-b	Graph-size / vocabulary controls	Balanced subsampling to R=25 recognized proteins (200 iter.)	0.955	10 / 10	n/a
S-c	Composition ablation	Removal of 18 Top-10-shared vocabulary proteins	0.999	9 / 10	n/a
S-c	Composition ablation	Removal of 18 random vocabulary proteins (20 iter., control)	—	10 / 10	n/a

Table S4. Disease specificity of the formulation ranking across three independent reference-drug models. The identical 56 formulation networks were scored under hypertension (HTN), type 2 diabetes (T2D), and hyperlipidemia (HLP) reference-drug models, each constructed from its corresponding disease-specific balanced reference dataset (HTN, 15 positive and 15 comparator drugs; T2D, 16 positive and 16 comparator drugs; HLP, 10 positive and 10 comparator drugs). Spearman ϱ compares mean SVM scores across all 56 formulations; Top-10 overlap indicates the number of formulations shared between the two Top-10 sets. The absence of overlap between the HTN and T2D rankings indicates that the prioritization is not determined by the structural properties of the candidate graphs alone. The partial overlap between HTN and HLP is consistent with shared cardiovascular drug-target space but cannot be fully separated from shared upstream selection characteristics.

**Comparison**	**Spearman rho (56 formulations)**	**Top-10 overlap**
HTN vs T2D	0.506	0/10
HTN vs HLP	0.901	7/10
T2D vs HLP	0.51	0/10

Table S5. Complete 110-protein stable SVM-derived antihypertensive-reference-associated signature with permutation calibration and PPI module assignment. Proteins satisfying the predefined directional-stability criteria across repeated out-of-fold (OOF) linear SVM models, ranked by mean HTN-versus-comparator OOF contribution difference. Encoded protein ID corresponds to the internal identifier used during modeling; STRING protein ID and Gene symbol were decoded after the signature was fixed. Empirical FDR and empirical q were derived from 1,000 label permutations using a threshold-specific false-discovery-rate estimate with a cumulative-minimum monotonicity correction. PPI module denotes the module assigned by weighted greedy modularity maximization (M1–M6). Module labels are descriptive annotations based on the predominant characteristics of member proteins and do not represent formal pathway-enrichment results.

Rank	Encoded protein ID	STRING protein ID	Gene symbol	OOF contribution difference	Median seed difference	Positive seed fraction	Positive coefficient fraction	Eligible models	Observed discoveries at threshold	Mean null discoveries	Empirical FDR	Empirical q	PPI module
1	3281	9606.ENSP00000272190	REN	0.0206	0.0206	1.00	1.00	50	1	0.111	0.111	0.099	M1
2	7084	9606.ENSP00000328968	SCN5A	0.0166	0.0166	1.00	1.00	50	2	0.306	0.153	0.099	M2
3	3774	9606.ENSP00000283254	SCN3A	0.0145	0.0145	1.00	1.00	50	3	0.557	0.186	0.099	M2
4	8837	9606.ENSP00000349320	CACNA2D1	0.0140	0.0140	1.00	1.00	50	4	0.610	0.153	0.099	M2
5	9510	9606.ENSP00000355192	CACNA1S	0.0131	0.0131	1.00	1.00	50	5	0.763	0.153	0.099	M2
6	14684	9606.ENSP00000398832	AGTR1	0.0130	0.0130	1.00	1.00	50	6	0.786	0.131	0.099	M1
7	2989	9606.ENSP00000266376	CACNA1C	0.0130	0.0130	1.00	1.00	50	7	0.791	0.113	0.099	M2
8	3997	9606.ENSP00000288139	CACNA1D	0.0130	0.0130	1.00	1.00	50	8	0.792	0.099	0.099	M2
9	5612	9606.ENSP00000309052	CATSPER1	0.0110	0.0110	1.00	1.00	50	9	1.363	0.151	0.126	M2
10	6414	9606.ENSP00000320025	CACNB2	0.0110	0.0109	1.00	1.00	50	10	1.372	0.137	0.126	M2
11	10663	9606.ENSP00000360973	AGTR2	0.0108	0.0108	1.00	1.00	50	11	1.416	0.129	0.126	M1
12	4109	9606.ENSP00000290866	ACE	0.0103	0.0103	1.00	1.00	50	12	1.652	0.138	0.126	M1
13	14020	9606.ENSP00000386306	SCN9A	0.0096	0.0096	1.00	1.00	50	13	1.950	0.150	0.126	M2
14	19531	9606.ENSP0000	SCN11	0.0096	0.0096	1.00	1.00	50	14	1.971	0.141	0.126	M2

		0499569	A										
15	14311	9606.ENSP00000390600	SCN10A	0.0095	0.0095	1.00	1.00	50	15	2.018	0.135	0.126	M2
16	8564	9606.ENSP00000346534	SCN8A	0.0095	0.0095	1.00	1.00	50	16	2.019	0.126	0.126	M2
17	16581	9606.ENSP00000441600	CCR3	0.0091	0.0090	1.00	1.00	50	17	2.315	0.136	0.131	M1
18	18675	9606.ENSP00000489829	CACNA1A	0.0087	0.0087	1.00	1.00	50	18	2.713	0.151	0.131	M2
19	10558	9606.ENSP00000360423	CACNA1B	0.0087	0.0087	1.00	1.00	50	19	2.735	0.144	0.131	M2
20	11497	9606.ENSP00000365441	CACNA1F	0.0087	0.0087	1.00	1.00	50	20	2.738	0.137	0.131	M2
21	9109	9606.ENSP00000352011	CACNA1G	0.0086	0.0086	1.00	1.00	50	21	2.791	0.133	0.131	M2
22	1520	9606.ENSP00000251127	NALCN	0.0085	0.0085	1.00	1.00	50	22	2.888	0.131	0.131	M2
23	9598	9606.ENSP00000355627	AGT	0.0082	0.0082	1.00	1.00	50	23	3.181	0.138	0.138	M1
24	11643	9606.ENSP00000366416	EDNRB	0.0074	0.0074	1.00	1.00	50	24	4.058	0.169	0.159	M1
25	11200	9606.ENSP00000363596	CNR2	0.0069	0.0069	1.00	1.00	50	25	4.771	0.191	0.159	M1
26	13849	9606.ENSP00000384932	GPR12	0.0069	0.0069	1.00	1.00	50	26	4.771	0.184	0.159	M3
27	10701	9606.ENSP00000361141	OPN4	0.0069	0.0069	1.00	1.00	50	28	4.771	0.170	0.159	M1
28	11110	9606.ENSP00000363136	GPR3	0.0069	0.0069	1.00	1.00	50	28	4.771	0.170	0.159	M3
29	12062	9606.ENSP00000369344	GPR150	0.0069	0.0069	1.00	1.00	50	29	4.781	0.165	0.159	M3
30	10584	9606.ENSP00000360538	FFAR4	0.0068	0.0068	1.00	1.00	50	30	4.913	0.164	0.159	M1
31	16231	9606.ENSP00000432456	GPR61	0.0068	0.0068	1.00	1.00	50	31	4.920	0.159	0.159	M3
32	16759	9606.ENSP00000445772	LTB4R2	0.0062	0.0062	1.00	1.00	50	32	6.088	0.190	0.181	M1
33	16665	9606.ENSP00000443256	LPAR2	0.0061	0.0060	1.00	1.00	50	33	6.287	0.191	0.181	M1
34	14252	9606.ENSP00000389326	ACE2	0.0061	0.0060	1.00	1.00	50	34	6.300	0.185	0.181	M1
35	14505	9606.ENSP00000394624	OPRM1	0.0061	0.0061	1.00	1.00	50	35	6.327	0.181	0.181	M1

36	16954	9606.ENSP00000451605	MC1R	0.0060	0.0060	1.00	1.00	50	36	6.525	0.181	0.181	M1
37	17006	9606.ENSP00000453076	GPR176	0.0058	0.0058	1.00	1.00	50	37	6.973	0.188	0.188	M3
38	7528	9606.ENSP00000334198	CACNA1H	0.0055	0.0055	1.00	1.00	50	38	7.894	0.208	0.200	M2
39	10443	9606.ENSP00000359793	PTGFR	0.0055	0.0055	1.00	1.00	50	40	8.017	0.200	0.200	M1
40	17811	9606.ENSP00000477548	HCRTR2	0.0055	0.0055	1.00	1.00	50	40	8.017	0.200	0.200	M1
41	10387	9606.ENSP00000359532	NTSR1	0.0054	0.0054	1.00	1.00	50	41	8.232	0.201	0.201	M1
42	10526	9606.ENSP00000360255	OPN5	0.0053	0.0053	1.00	1.00	50	42	8.575	0.204	0.204	M4
43	13002	9606.ENSP00000377948	QRFPR	0.0046	0.0046	1.00	1.00	50	44	11.441	0.260	0.249	M1
44	13100	9606.ENSP00000378548	GPR135	0.0046	0.0046	1.00	1.00	50	44	11.441	0.260	0.249	M3
45	17200	9606.ENSP00000458791	GPR139	0.0046	0.0046	1.00	1.00	50	45	11.573	0.257	0.249	M3
46	13779	9606.ENSP00000384387	HCRTR1	0.0046	0.0046	1.00	1.00	50	46	11.608	0.252	0.249	M1
47	756	9606.ENSP00000226021	CACNG1	0.0045	0.0046	1.00	1.00	50	47	11.878	0.253	0.249	M2
48	12117	9606.ENSP00000369643	GRPR	0.0045	0.0045	1.00	1.00	50	48	11.941	0.249	0.249	M1
49	11169	9606.ENSP00000363431	NPY4R	0.0044	0.0044	1.00	1.00	50	49	12.654	0.258	0.257	M1
50	12453	9606.ENSP00000371927	GPR78	0.0044	0.0043	1.00	1.00	50	50	12.837	0.257	0.257	M3
51	14280	9606.ENSP00000389814	ADIPOQ	0.0043	0.0043	1.00	1.00	50	51	13.122	0.257	0.257	M1
52	11966	9606.ENSP00000368683	EDN1	0.0041	0.0042	1.00	1.00	50	52	14.347	0.276	0.276	M1
53	13975	9606.ENSP00000386069	ADRA2C	0.0041	0.0041	1.00	1.00	50	53	14.615	0.276	0.276	M1
54	19586	9606.ENSP00000501180	MAS1	0.0040	0.0040	1.00	1.00	50	54	15.667	0.290	0.290	M1
55	14368	9606.ENSP00000391826	IGF2	0.0037	0.0046	0.80	1.00	48	55	18.282	0.332	0.317	M2
56	9809	9606.ENSP00000356658	GPR52	0.0036	0.0037	1.00	1.00	50	56	18.982	0.339	0.317	M3
57	9939	9606.ENSP0000	RXFP4	0.0036	0.0037	1.00	1.00	50	57	18.982	0.333	0.317	M1

		0357301											
58	5461	9606.ENSP00000307240	KRT74	0.0036	0.0037	1.00	1.00	50	59	19.027	0.322	0.317	M5
59	3036	9606.ENSP00000267119	KRT71	0.0036	0.0037	1.00	1.00	50	59	19.027	0.322	0.317	M5
60	10425	9606.ENSP00000359682	BRS3	0.0036	0.0036	1.00	1.00	50	60	19.536	0.326	0.317	M1
61	10414	9606.ENSP00000359643	LPAR3	0.0036	0.0036	1.00	1.00	50	61	19.546	0.320	0.317	M1
62	9727	9606.ENSP00000356251	GPR37L1	0.0036	0.0036	1.00	1.00	50	62	19.837	0.320	0.317	M1
63	10224	9606.ENSP00000358783	NPBWR2	0.0036	0.0036	1.00	1.00	50	63	19.993	0.317	0.317	M1
64	10272	9606.ENSP00000358967	OPN1LW	0.0035	0.0035	1.00	1.00	50	65	20.615	0.317	0.317	M4
65	17628	9606.ENSP00000472316	OPN1MW	0.0035	0.0035	1.00	1.00	50	65	20.615	0.317	0.317	M4
66	12311	9606.ENSP00000370950	NPSR1	0.0034	0.0035	1.00	1.00	50	66	21.576	0.327	0.318	M1
67	9151	9606.ENSP00000352427	RGR	0.0034	0.0035	1.00	1.00	50	67	21.595	0.322	0.318	M4
68	9571	9606.ENSP00000355512	OPN3	0.0034	0.0035	1.00	1.00	50	68	21.595	0.318	0.318	M4
69	4535	9606.ENSP00000296658	CMBL	0.0029	0.0029	1.00	1.00	50	69	26.252	0.380	0.377	M1
70	11977	9606.ENSP00000368766	ADRA1D	0.0029	0.0026	1.00	1.00	50	70	26.706	0.382	0.377	M1
71	14094	9606.ENSP00000386884	CXCR4	0.0028	0.0026	1.00	1.00	50	71	26.910	0.379	0.377	M1
72	5436	9606.ENSP00000307014	KRT73	0.0028	0.0028	1.00	1.00	50	73	27.501	0.377	0.377	M5
73	4249	9606.ENSP00000293745	KRT72	0.0028	0.0028	1.00	1.00	50	73	27.501	0.377	0.377	M5
74	8318	9606.ENSP00000343782	ADRB3	0.0026	0.0026	1.00	1.00	50	74	29.463	0.398	0.393	-
75	13369	9606.ENSP00000380432	INS	0.0025	0.0028	0.90	1.00	49	75	30.902	0.412	0.393	M2
76	3735	9606.ENSP00000282499	GRIA4	0.0025	0.0027	0.90	1.00	49	76	30.956	0.407	0.393	M6
77	11496	9606.ENSP00000365437	GAD2	0.0025	0.0031	0.80	1.00	48	77	30.983	0.402	0.393	M6
78	4516	9606.ENSP00000296526	GRIA2	0.0025	0.0027	0.90	1.00	49	78	31.343	0.402	0.393	M2

79	18174	9606.ENSP00000481554	GRIA3	0.0025	0.0027	0.90	1.00	49	79	31.411	0.398	0.393	M6
80	16435	9606.ENSP00000437737	HTR2A	0.0025	0.0027	1.00	1.00	50	80	31.478	0.393	0.393	M1
81	13862	9606.ENSP00000385019	CACNA1I	0.0024	0.0031	0.80	1.00	48	81	31.865	0.393	0.393	M2
82	6379	9606.ENSP00000319591	KCND3	0.0024	0.0026	0.90	1.00	49	82	32.432	0.396	0.396	M2
83	11191	9606.ENSP00000363553	LPAR1	0.0023	0.0023	1.00	1.00	50	83	33.635	0.405	0.400	M1
84	9399	9606.ENSP00000354652	NPY1R	0.0023	0.0023	1.00	1.00	50	84	33.636	0.400	0.400	M1
85	5924	9606.ENSP00000312652	LEP	0.0022	0.0024	0.90	1.00	49	85	34.612	0.407	0.407	M1
86	8937	9606.ENSP00000350256	CCR9	0.0017	0.0017	1.00	1.00	50	86	40.136	0.467	0.447	M1
87	11526	9606.ENSP00000365663	NPPA	0.0017	0.0019	0.90	1.00	49	87	40.432	0.465	0.447	M1
88	8932	9606.ENSP00000350198	SSTR2	0.0017	0.0016	1.00	1.00	50	88	40.809	0.464	0.447	M1
89	15863	9606.ENSP00000423917	NPY5R	0.0017	0.0016	1.00	1.00	50	89	40.845	0.459	0.447	M1
90	15409	9606.ENSP00000415786	SERPINE2	0.0016	0.0020	0.80	1.00	48	90	41.332	0.459	0.447	M1
91	16264	9606.ENSP00000433208	PTH	0.0016	0.0018	0.90	1.00	49	91	41.821	0.460	0.447	M1
92	2356	9606.ENSP00000262209	TRPA1	0.0016	0.0020	0.80	1.00	48	92	41.838	0.455	0.447	M2
93	15557	9606.ENSP00000418081	CACNA2D2	0.0015	0.0017	0.90	1.00	49	93	42.988	0.462	0.447	M2
94	12991	9606.ENSP00000377840	CACNB1	0.0015	0.0018	0.80	1.00	48	94	43.084	0.458	0.447	M2
95	16817	9606.ENSP00000447378	MGAM	0.0014	0.0015	0.90	1.00	49	95	44.144	0.465	0.447	M1
96	19538	9606.ENSP00000499717	CALM1	0.0013	0.0014	1.00	1.00	50	96	44.869	0.467	0.447	M2
97	18095	9606.ENSP00000480573	ADRA2B	0.0012	0.0013	1.00	1.00	50	97	45.098	0.465	0.447	M1
98	670	9606.ENSP00000223095	SERPINE1	0.0012	0.0013	1.00	1.00	50	98	45.128	0.460	0.447	M1
99	6440	9606.ENSP00000320295	TUBB3	0.0012	0.0012	0.90	1.00	50	99	45.205	0.457	0.447	M1
100	13345	9606.ENSP0000	GPER1	0.0011	0.0011	1.00	1.00	50	100	45.874	0.459	0.447	M1

		0380281											
101	11624	9606.ENSP0000 0366307	THBD	0.0011	0.0013	0.80	1.00	48	101	46.165	0.457	0.447	M1
102	19544	9606.ENSP0000 0499797	CALM2	0.0011	0.0013	1.00	1.00	50	102	46.262	0.454	0.447	M2
103	12168	9606.ENSP0000 0369960	ADRA1 A	0.0010	0.0011	1.00	1.00	50	103	46.267	0.449	0.447	M1
104	10175	9606.ENSP0000 0358513	CNR1	0.0010	0.0008	1.00	1.00	50	104	46.813	0.450	0.447	M1
105	12522	9606.ENSP0000 0373169	DRD3	0.0008	0.0006	0.80	1.00	50	105	47.673	0.454	0.447	M1
106	7902	9606.ENSP0000 0339191	CAV1	0.0008	0.0012	0.90	1.00	50	106	47.694	0.450	0.447	M2
107	9691	9606.ENSP0000 0356094	AVPR1 B	0.0005	0.0003	0.80	0.98	50	107	48.870	0.457	0.447	M1
108	6075	9606.ENSP0000 0315011	EDNRA	0.0005	0.0004	0.80	1.00	50	108	48.926	0.453	0.447	M1
109	19351	9606.ENSP0000 0497931	ABCB1 1	0.0005	0.0009	0.80	1.00	50	109	48.992	0.449	0.447	-
110	9443	9606.ENSP0000 0354859	DRD2	0.0004	0.0005	0.90	0.96	50	110	49.146	0.447	0.447	M1

No individual protein reached strict empirical $q < 0.05$. Eight proteins reached exploratory $q < 0.10$; mechanistic inference therefore emphasized stable network/module-level structure rather than single-protein significance.

Table S6. Molecular coverage of the stable hypertension signature across all 56 insurance-covered Korean herbal formulations. *Network nodes* denotes the number of proteins retained in each formulation network after applying the STITCH combined-score selection rule (top 100 proteins per formulation; see Methods). *Stable signature overlap* is the number of the 110 stable SVM-derived proteins present in the formulation network; *weighted core coverage* weights each overlapping protein by its mean OOF contribution difference. *M1* and *M2 coverage* apply the same weighting restricted to the corresponding module denominators.

Coverage rank	Formulation	Network nodes	Stable signature overlap (n)	Weighted core coverage (%)	M1 overlap (n)	M1 coverage (%)	M2 overlap (n)	M2 coverage (%)	Coverage percentile
1	Daecheongryong-tang	100	22	15.2	21	34.0	1	0.7	100.0
2	Daehwangmokdanpi-tang	100	21	14.9	21	34.0	0	0.0	98.2
3	Doinseunggi-tang	100	21	14.9	21	34.0	0	0.0	96.4
4	Hyangsapyeongwi-san	99	21	14.9	21	34.0	0	0.0	94.5
5	Sigyeongbanha-tang	100	20	14.6	20	33.3	0	0.0	92.7
6	Haengso-tang	100	20	14.6	20	33.3	0	0.0	90.9
7	Antae-eum	98	20	14.6	20	33.3	0	0.0	89.1
8	Daehwajung-eum	98	14	11.3	14	25.9	0	0.0	87.3
9	Naeso-san	97	12	10.5	12	24.0	0	0.0	85.5
10	Samchulgeonbi-tang	98	12	10.5	12	24.0	0	0.0	83.6
11	Socheongryong-tang	99	6	5.9	5	12.9	1	0.7	81.8
12	Daesiho-tang	98	5	5.5	5	12.7	0	0.0	80.0
13	Jowiseunggi-tang	92	5	5.5	5	12.6	0	0.0	78.2
14	Samhwangsasim-tang	94	4	5.3	4	12.2	0	0.0	76.4
15	Gamisoyo-san	96	3	4.8	2	10.3	1	0.7	74.5
16	Yeongyopaedok-san	93	3	4.8	2	10.3	1	0.7	72.7
17	Sihosogan-tang	98	3	4.8	2	10.3	1	0.7	70.9
18	Sihogyeji-tang	97	3	4.8	2	10.3	1	0.7	69.1
19	Danggwiyukhwang-tang	94	3	4.7	3	10.8	0	0.0	67.3
20	Banhasasim-tang	94	2	4.5	2	10.3	0	0.0	65.5
21	Hwangnyeonhaedok-tang	94	2	4.5	2	10.3	0	0.0	63.6
22	Gungha-tang	94	3	4.2	2	9.0	1	0.7	61.8
23	Ijung-tang	90	3	4.2	2	9.0	1	0.7	60.0
24	Ijin-tang	93	3	4.2	2	9.0	1	0.7	58.2
25	Sihocheonggan-tang	96	2	3.9	2	9.0	0	0.0	56.4
26	Sosiho-tang	96	2	3.9	2	9.0	0	0.0	54.5
27	Yikwiseungyang-tang	97	2	3.9	2	9.0	0	0.0	52.7
28	Insampaedok-san	97	2	3.9	2	9.0	0	0.0	50.9

29	Hwanggeumjakyak-tang	97	2	3.9	2	9.0	0	0.0	49.1
30	Gumi-ganghwal-tang	96	2	3.9	2	9.0	0	0.0	47.3
31	Cheongsanggyeontong-tang	97	2	3.9	2	9.0	0	0.0	45.5
32	Orim-san	97	2	3.9	2	9.0	0	0.0	43.6
33	Samhojagyak-tang	96	2	3.9	2	9.0	0	0.0	41.8
34	Samso-eum	96	2	3.9	2	9.0	0	0.0	40.0
35	Pyeongwi-san	91	2	3.9	2	9.0	0	0.0	38.2
36	Palmul-tang	92	2	3.9	2	9.0	0	0.0	36.4
37	Bulhwangeumjeonggi-san	97	2	3.9	2	9.0	0	0.0	34.5
38	Bojungikgi-tang	94	2	3.9	2	9.0	0	0.0	32.7
39	Boheo-tang	96	2	3.9	2	9.0	0	0.0	30.9
40	Baekchul-tang	95	2	3.9	2	9.0	0	0.0	29.1
41	Hyeonggaeyeongyo-tang	98	1	3.7	1	8.5	0	0.0	27.3
42	Danggwiyeongyo-eum	98	1	3.7	1	8.5	0	0.0	25.5
43	Ojeok-san	98	5	2.2	4	4.4	1	0.7	23.6
44	Injinho-tang	91	3	1.6	3	3.7	0	0.0	21.8
45	Seungyangbowi-tang	100	3	1.5	2	2.9	1	0.7	20.0
46	Galgeun-tang	100	3	1.5	2	2.9	1	0.7	18.2
47	Galgeunhaegi-tang	99	2	1.3	2	2.9	0	0.0	16.4
48	Hoechunyanggyeok-san	96	2	1.1	1	1.8	1	0.7	14.5
49	Jaeumganghwa-tang	94	1	0.8	1	1.8	0	0.0	12.7
50	Banhabaekchulcheonma-tang	94	1	0.8	1	1.8	0	0.0	10.9
51	Cheongwi-san	90	1	0.8	1	1.8	0	0.0	9.1
52	Gungso-san	99	1	0.5	1	1.2	0	0.0	7.3
53	Bokryeongbosim-tang	100	1	0.5	1	1.2	0	0.0	5.5
54	Cheongseoikgi-tang	99	1	0.5	1	1.2	0	0.0	3.6
55	Banhahubak-tang	75	1	0.3	0	0.0	1	0.7	1.8
56	Saengmaek-san	85	1	0.2	1	0.4	0	0.0	0.0

Table S7. Decoded blind GCN Top-15 protein-identity attribution and cross-model network metrics. Attribution was performed on three representative high-priority formulation networks (Antae-eum, Hyangsapyeongwi-san, Sigyeongbanha-tang) using identity occlusion, in which each protein's encoded identity was replaced by the out-of-vocabulary identity while retaining its numerical node features and PPI connectivity. Protein identities remained encoded throughout attribution and ranking and were decoded to gene symbols only after the Top-15 set had been fixed. Δ logit denotes the reduction in the GCN hypertension prediction score upon identity masking. SVM stable overlap and SVM module indicate membership in the 110-protein stable SVM signature and its assigned module. GCN attribution is formulation-centric; these metrics reflect internal cross-model convergence within representative candidate graphs rather than independent disease-wide validation.

GCN rank	Encoded protein ID	Gene symbol	Mean delta logit	Median delta logit	Positive seed fraction	Top-20% seed fraction	Training prevalence difference	GCN residual z	Selected formulation occurrence (n)	SVM stable overlap	SVM module	Candidate-network weighted degree	Cross-model class
1	9598	AGT	1.008	1.052	0.90	0.70	0.53	1.383	3	Yes	M1	30	Shared with stable SVM M1
2	10663	AGTR2	0.742	0.738	0.80	0.70	0.47	0.939	3	Yes	M1	27	Shared with stable SVM M1
3	15863	NPY5R	0.383	0.448	0.80	0.70	0.13	2.881	1	Yes	M1	6	Shared with stable SVM M1
4	16581	CCR3	0.297	0.369	0.80	0.70	0.40	0.268	3	Yes	M1	15	Shared with stable SVM M1
5	14094	CXCR4	0.517	0.778	0.70	0.70	0.33	2.332	3	Yes	M1	26	Shared with stable SVM M1
6	19475	CYP3A4	0.297	0.278	0.70	0.65	0.20	0.320	2	No		4	GCN-attributed only

7	17219	TRPV1	0.083	0.202	0.70	0.65	-0.07	1.187	2	No		6	GCN-attributed only
8	9443	DRD2	0.368	0.469	0.80	0.63	0.27	1.196	3	Yes	M1	12	Shared with stable SVM M1
9	13176	CXCL1	0.289	0.154	0.80	0.63	-0.07	3.057	3	No		15	GCN-attributed only
10	11200	CNR2	0.258	0.277	0.70	0.63	0.33	0.360	3	Yes	M1	8	Shared with stable SVM M1
11	11169	NPY4R	0.208	0.214	0.70	0.63	0.33	-0.102	3	Yes	M1	12	Shared with stable SVM M1
12	12522	DRD3	0.353	0.205	0.90	0.57	0.33	0.967	3	Yes	M1	12	Shared with stable SVM M1
13	10746	MMP9	0.114	0.207	0.70	0.57	-0.13	1.669	3	No		14	GCN-attributed only
14	19079	KNG1	0.255	0.247	0.60	0.55	0.07	1.512	2	No		15	GCN-attributed only
15	14505	OPRM1	0.143	0.294	0.70	0.53	0.40	-2.310	3	Yes	M1	22	Shared with stable SVM M1

Table S8. Comparison of the formulation ranking with independently measured vasorelaxant activity. The 22 formulations common to the present 56-formulation library and the published screen of 50 traditional herbal prescriptions on isolated rat aortic rings are listed in order of their rank in the present model.

Formulation	THP ID in Kim et al. 2020	SVM rank (of 56)	Mean SVM decision score	Relaxation at 1000 μg/mL (%)	SEM	Significant relaxation vs control	P vs control
**Hyangsapyeongwi-san**	THP46	4	0.3266	44.2	7.7	Yes	P < 0.01
**Naeso-san**	THP47	9	-0.0227	40.0	12.0	Yes	P < 0.05
**Samchulgeonbi-tang**	THP6	10	-0.0687	14.2	6.7	Yes	P < 0.05
**Saengmaek-san**	THP35	11	-0.1454	21.7	6.1	Yes	P < 0.01
**Hwangnyeonhaedok-tang**	THP17	15	-0.2717	43.7	7.4	Yes	P < 0.01
**Insampaedok-san**	THP12	18	-0.2939	28.0	6.0	Yes	P < 0.01
**Banhasasim-tang**	THP33	22	-0.3147	91.7	2.7	Yes	P < 0.01
**Jaeumganghwa-tang**	THP1	31	-0.3522	9.6	8.3	No	n.s.
**Banhabaekchulcheonma-tang**	THP9	33	-0.356	33.7	7.5	Yes	P < 0.01
**Gumi-ganghwal-tang**	THP13	34	-0.3571	11.6	3.2	No	n.s.
**Galgeun-tang**	THP2	36	-0.3641	13.2	4.5	No	n.s.
**Ojeok-san**	THP4	39	-0.3828	13.3	10.0	No	n.s.

**Sosiho-tang**	THP23	40	-0.3958	-0.6	10.0	No	n.s.
**Socheongryong-tang**	THP11	41	-0.4186	30.0	5.5	Yes	P < 0.05
**Ijung-tang**	THP16	44	-0.5041	16.2	4.0	Yes	P < 0.01
**Ijin-tang**	THP8	46	-0.5121	4.0	5.2	No	n.s.
**Samso-eum**	THP25	48	-0.5551	-16.0	10.5	No	n.s.
**Gamisoyo-san**	THP40	49	-0.5567	43.3	7.0	Yes	P < 0.05
**Bulhwangeumjeonggi-san**	THP50	50	-0.5627	14.2	2.5	Yes	P < 0.05
**Pyeongwi-san**	THP15	52	-0.5702	19.7	5.3	Yes	P < 0.01
**Palmul-tang**	THP5	53	-0.5714	7.6	4.0	No	n.s.
**Bojungikgi-tang**	THP10	55	-0.6087	5.0	5.1	No	n.s.
